# Characteristics of rhizosphere and endogenous bacterial community of Ulleung-sanmaneul, an endemic plant in Korea: application for alleviating salt stress

**DOI:** 10.1038/s41598-022-25731-z

**Published:** 2022-12-07

**Authors:** Swarnalee Dutta, Yeong-Su Kim, Yong Hoon Lee

**Affiliations:** 1 Division of Biotechnology, Jeonbuk National University, 79 Gobong-ro, Iksan-si, Jeollabuk-do 54596 Republic of Korea; 2 Wild Plants and Seeds Conservation Department, Baekdudaegan National Arboretum, Bonghwa-gun, Gyeongsangbuk-do 36209 Republic of Korea; 3 Advanced Institute of Environment and Bioscience, Plant Medical Research Center, and Institute of Bio-Industry, Jeonbuk National University, Jeonju-si, Republic of Korea

**Keywords:** Microbiology, Molecular biology, Plant sciences, Environmental sciences

## Abstract

Microbes influence plant growth and fitness. However, the structure and function of microbiomes associated with rare and endemic plants remain underexplored. To investigate the bacterial community structure of Ulleung-sanmaneul (U-SMN), an endemic plant in Korea, samples were collected from natural and cultivated habitats, and their 16S rDNA was sequenced. The root bacterial community structure differed from those of bulk soil and rhizosphere in both habitats. Endogenous bacteria in cultivated plants were less diverse than wild plants, but *Luteibacter rhizovicinus*, *Pseudomonas fulva*, and *Sphingomonas pruni* were shared. Co-inoculation of *Pseudoxanthomonas* sp. JBCE485 and *Variovorax paradoxus* JBCE486 promoted growth and induced salt stress resistance in Arabidopsis and chive. Changes in growth promotion and phenotypes of plants by co-inoculation were mediated by increased auxin production. Each strain colonized the roots without niche competition. The results indicated that host selectivity was influential than environmental factors in formulating endophytic bacterial composition, and domestication simplified the bacterial community diversity. Our results will contribute to the growth and maintenance of endemic U-SMN plants.

## Introduction

**Figure 1:**
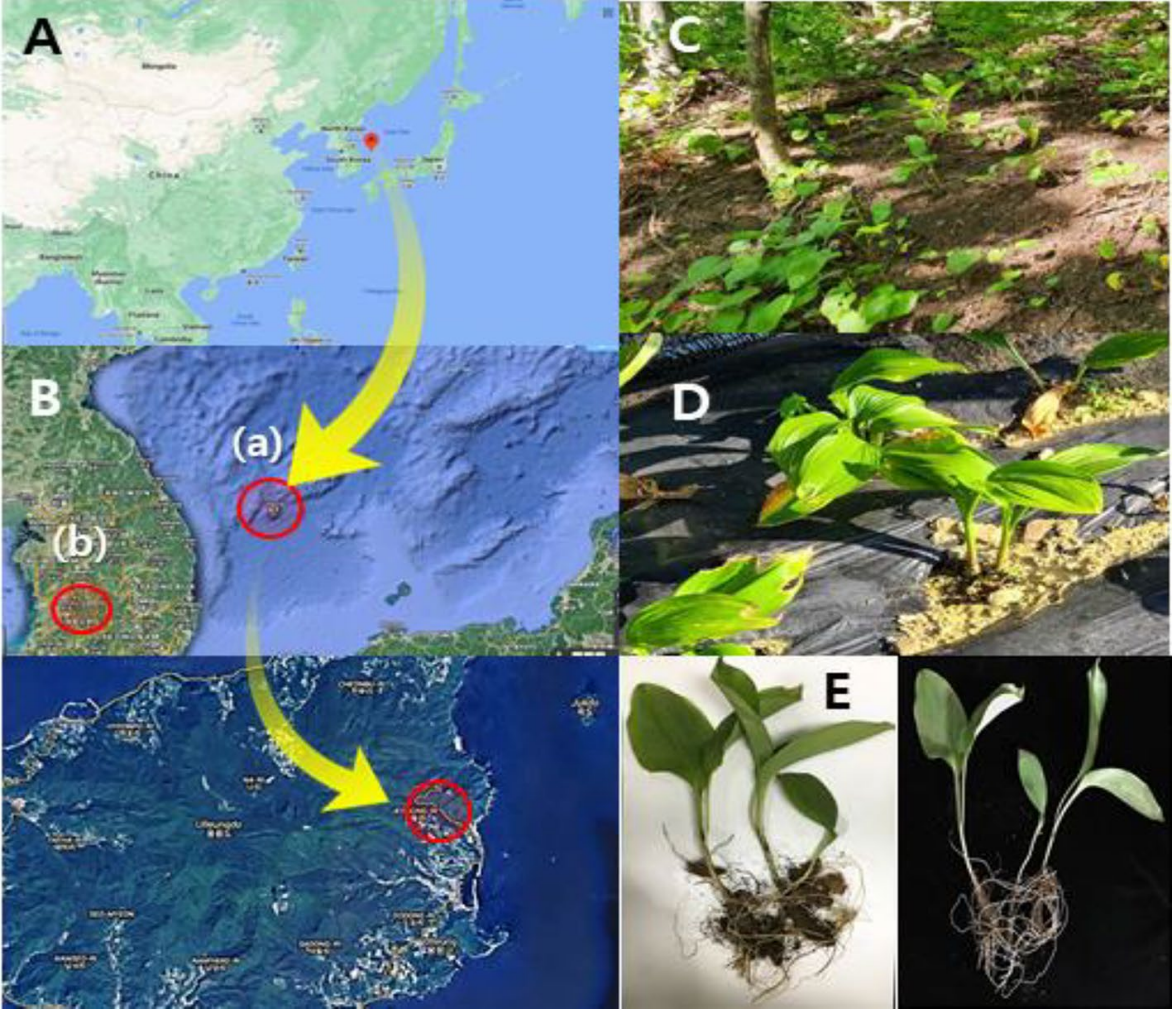
Sampling sites and Ulleung-sanmaneul plants. The endemic plants collected from native habitats and cultivated fields located (red dot) in Ulleung island, Gyeongsangbuk-do (**A,B-a,C**) and a medicinal herb experiment station, Jinan-gun, Jeollabuk-do (**B-b,D**), respectively. The plants (**E**) collected from native (left) and cultivated (right) areas for microbe profiling. The images (**A,B**) were obtained from https://www.google.co.kr/maps on July 2020.

*Allium ochotense* and *Allium microdictyon* (Liliaceae) are commonly known as Sanmaneul (mountain garlic) or Siberian onion and are distributed in many countries such as Korea, China, and Mongolia^1,2^. Their leaves and bulbs have been used not only as wild edible herbs but also as traditional medicines because of their anti-cancer, antioxidant, and antiatherogenic activities^3,4^. In Korea, these two species had been at risk of extinction due to the damage to their natural habitat, but recently they have been widely cultivated in Korea as an edible plant named Myeong-i-na-mul. *Allium ulleungense* H. J. Choi & N. Friesen (subg. Anguinum, Amaryllidaceae) is a new endemic species in South Korea, which is called Ulleung-sanmaneul (U-SMN). This species is mainly distributed on Ulleung Island, South Korea, as indicated by its name (Fig. 1). It is clearly distinguished from its close relatives, *A. microdictyon* and *A. ochotense,* by its broader leaves and larger whitish perianth and diploid chromosome numbers. In addition, molecular phylogenetic analyses using nuclear and chloroplast markers indicated that *A. ulleungense* is genetically distinct from other species of the subg. Anguinum, Amaryllidaceae^5^. Oceanic islands of volcanic origin (like Ulleung Island) are vulnerable and susceptible to climate change, natural catastrophes, saline intrusion and overexploitation of natural resources^6^. Moreover, demand as an edible crop makes endemic species more vulnerable to anthropogenic threats and natural changes^7^. The restricted distribution of U-SMN poses additional attention for means of conservation.

Conservation approaches, including in situ conservation and biotechnological approaches, have been implemented for endemic species^8^. However, conservation efforts require detailed studies on plant biology, dynamics, and factors affecting the growth and adaptation of endemic plants^9^. Cultivation of rare endemic plants also requires a thorough understanding of their ecology and factors affecting their growth and propagation. Mutualistic microbes that have adapted to associate with plants can enhance ecosystem functioning and restoration, as well as promote plant growth and fitness^10^. Plant associated microbial communities are indicated as essential factors to be considered for conservation approaches^11^. Domestication or cultivation of endemic plants for conservation yields desired results when natural conditions suitable for the plant, including the associated microbiome, are maintained during cultivation^12^. However, adequate information on assemblage of microbial community structure and diversity, and their influence on the host plant is limited.

Bacteria associated with plants can be potentially beneficial. The application of beneficial microbes can increase biomass and fitness of plants with minimum input of pesticides and fertilizers, and some of the selected microbes have been formulated and developed as commercial products^13,14^. Microbes that reside around *Allium* spp. have been used as biocontrol agents or biofertilizers. The antagonistic bacterium *Streptomyces* sp. isolated from Welsh onion (*Allium fistulosum*) and Chinese leek inhibits the infection of Chinese cabbage by *Alternaria brassicicola*^15,16^. The accumulation of antagonistic *Flavobacterium* sp. in the soil cultivating *Allium* suppresses Fusarium wilt, which emphasizes the need to analyze the recruitment of bacteria by *Allium* plants for eco-friendly management of diseases^17^. *Lactobacillus fermentum* isolated from *Allium ursinum* leaves successfully survive simulated gastrointestinal conditions and show a protective immunomodulatory effect that can be beneficial if used as a probiotic for humans^18^. However, there is limited information about microbiome of overall Ulleungdo islands^19^ and, to the best of our knowledge, bacterial community compositions and functions of U-SMN have not yet been explored till date.

In this study, we analyzed the 16S rDNA sequencing data of rhizospheric and endophytic bacterial communities of U-SMN plants collected from the Ulleung Island, Korea, where the plant is endemic, and compared the bacterial compositions with those of cultivated U-SMN plants. In addition, we isolated and selected potent bacterial strains that promoted plant growth and induced salt stress resistance in *Allium* sp. The major constraints for successful application of potential strains under field condition have been attributed to the limited establishment of the inoculant, insufficient competition, or inadequate compatibility of the inoculant with hosts and the indigenous microbial community^20–22^. Our study indicated that co-inoculation of the selected bacterial strains promoted the plant growth, and induced salt stress resistance in the plants.

## Results

### Sampling of U-SMN plants and soil properties of the habitat

Microbes that inhabit the inside or surface of plants influence their productivity and health. To study the microbial community structure associated with various rhizocompartments (bulk soil, rhizosphere soil, and root endosphere) of U-SMN plants, we collected roots and surrounding soils from wild habitats (Fig. 1A–C) and cultivated fields (Fig. 1B,D) in July 2020.

The soils of the wild area were slightly acidic (< pH 6.0), which is characteristic of Ulleung Island, that is the endemic habitat of the plants. The soils in the cultivated area were around neutral or weakly acidic pH (> pH6.8) and had higher calcium, potassium, and sodium contents compared to soils from wild areas (Supplementary Table S1).

### 16S rDNA sequencing data associated with U-SMN plants

The bacterial microbiota associated with U-SMN plants in the wild and cultivated areas were analyzed by 16S rDNA sequencing. A total of 1,231,122 (median: 68,396) bacterial 16S rRNA reads were generated through high-throughput sequencing, and 703,604 reads with a median read length of 413.2, were produced after quality trimming, merging, and removal of chimeric reads. Bulk soil and rhizosphere showed an average of 5321 and 5631 operational taxonomic units (OTUs), respectively, in the wild area; and 4806 and 5086 OTUs, respectively, in the cultivated area (Supplementary Tables S2, S3) at a 3% dissimilarity cut-off for the rarefaction curve (Supplementary Fig. S1). However, the average number of OTUs from the roots of wild and cultivated areas were 119 and 157, respectively. The Good’s coverage analysis revealed more than 98.57% of the taxonomic richness was covered by sequencing efforts in all the samples of wild and cultivated areas, excluding more than 94% of richness in root samples from wild areas (Supplementary Tables S2, S3). Considering many previous reports^23^, we concluded that our data were sufficient to compare the changes in bacterial diversity between bulk soil, rhizosphere, and roots. For the sequencing data of bulbs, an average of 83 OTUs from three cultivated area samples and 45 OTUs from one wild area sample were obtained. Because of low reads in biological replications of wild samples, data were not included for the overall analysis and were used for the analysis of the presence or absence of specific species.

### Bacterial diversities by alpha diversity indices

The bacterial diversities in bulk soil and rhizosphere were higher than those in roots in both native and cultivated areas based on non-parametric analysis of diversity indices such as ACE and Chao1 (Supplementary Tables S2, S3). There was no significant difference between the bacterial diversity of bulk soil and the rhizosphere of wild and cultivated areas, despite the differences in soil chemical properties. Similar results were recorded for the Shannon and phylogenetic diversity indices, which also indicated a greater abundance and richness of bacterial communities in bulk soil and rhizosphere than in root endophytes (Supplementary Tables S2, S3) in both wild and cultivated areas. Taken together, the bacterial diversities were maintained at higher levels in the bulk soil and rhizosphere than in the endophyte, irrespective of habitat. There were no significant differences in bacterial diversity between the compartments of the wild and cultivated habitats.

### Difference of bacterial community structure by beta diversity analysis

**Figure 2:**
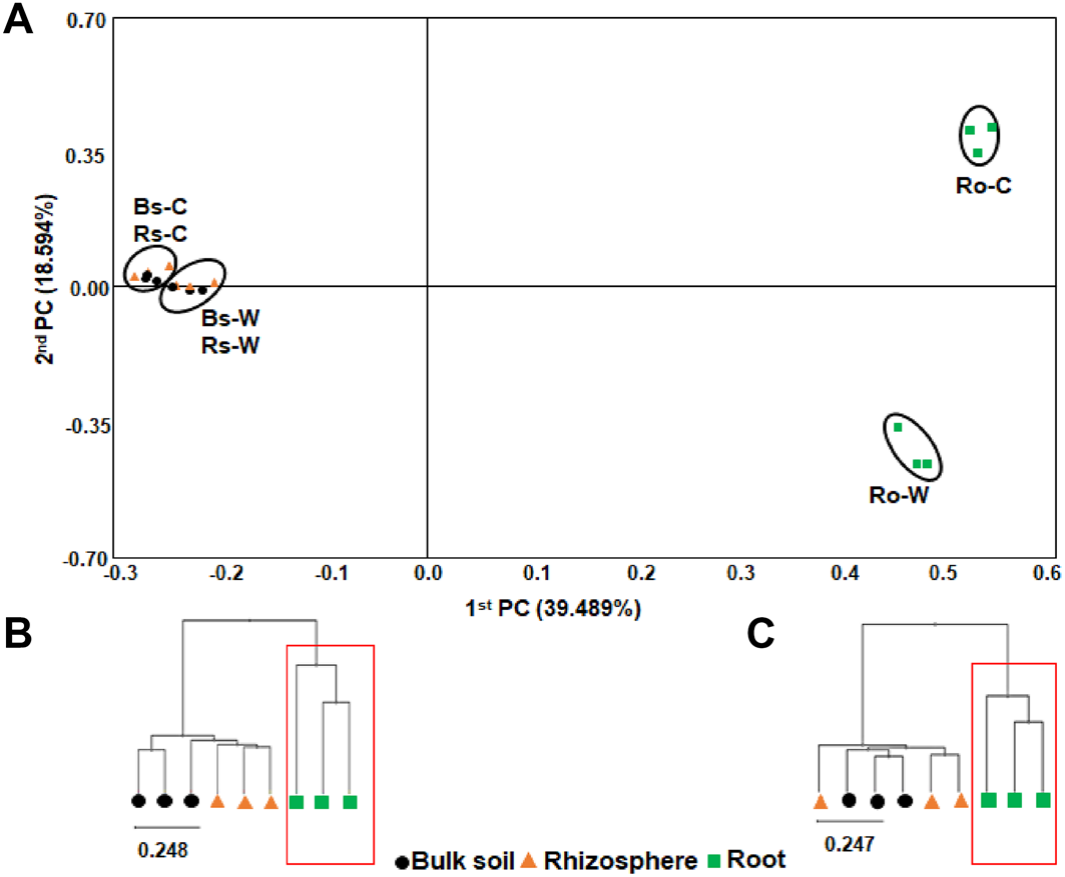
Principal coordinates analysis (PCoA) and UPGMA clustering of bacterial composition. (**A**) PCoA and UPGMA clustering based on Bray–Curtis dissimilarity in (**B**) wild and (**C**) cultivated areas using relative abundances of all OTUs at the species level. The red box shows the cluster formed exclusively in roots of both wild and cultivated habitats.

PCoA analysis indicated that the bacterial taxonomic structure and composition in the bulk soil and rhizosphere soil did not differ significantly between the wild and cultivated areas (Fig. 2A). The bacterial diversities of the bulk soil and the rhizosphere in both habitats, although clustered separately, were very similar. The bacterial structure of the roots at the genus and species levels formed a distinct cluster compared to the bulk soil and rhizosphere in both wild and cultivated areas (Fig. 2A). UPGMA clustering also showed that while the bacterial community structures of the bulk soil and rhizosphere in each habitat were clustered together, the bacterial community structures of roots formed a distinct cluster from the soils (Fig. 2B,C), indicating the uniqueness of the bacterial composition of endophytes. Taken together, the results revealed that the bacterial community structures of the roots were significantly different from those of the bulk soil and rhizosphere, indicating that the selectivity of host plants is more influential than environmental factors for endophytic populations in each habitat.

### Taxonomic structure of bacteria at the phylum level

**Figure 3:**
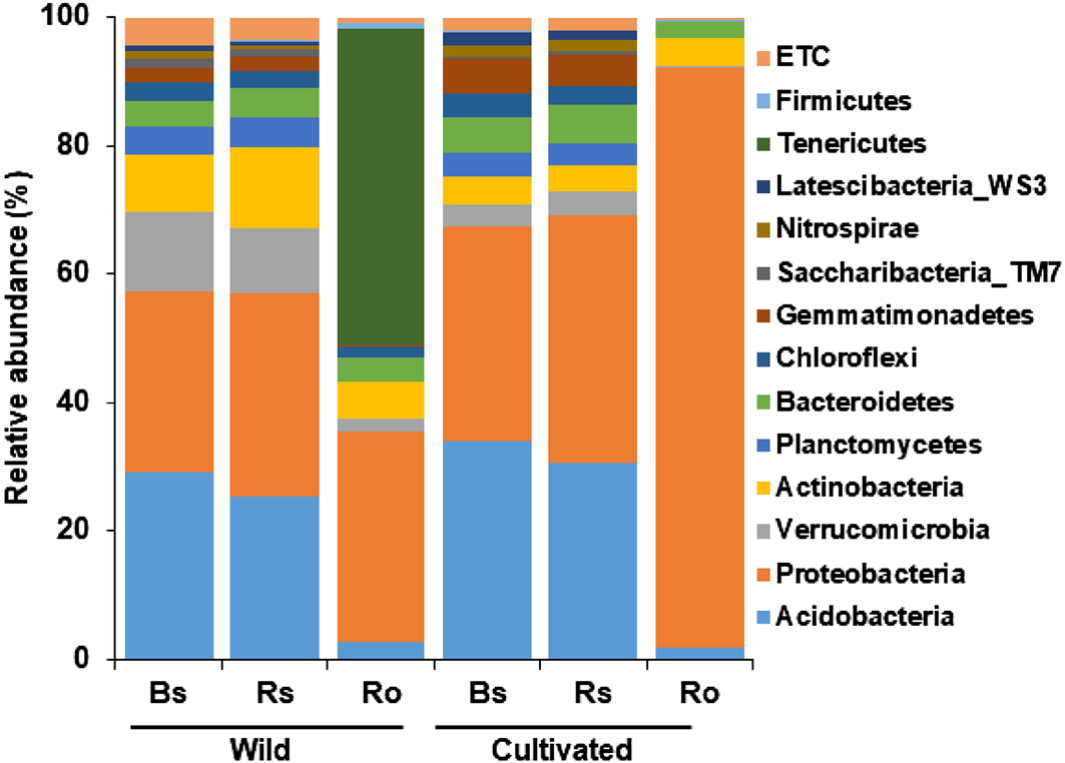
Comparative analysis of bacterial composition and relative abundance at the phylum level. The bulk soil (Bs), rhizosphere soil (Rs), and root (Ro) samples of Ulleung-sanmaneul plants collected from wild and cultivated areas. Phyla with relative abundances more than 1% in at least one compartment were selected and compared. Phyla with relative abundances less than 1% were referred to as “ETC”.

Comparison of bacterial profiles at the phylum level between rhizocompartments from wild and cultivated U-SMN plants showed that the bacterial compositions of bulk soil and rhizosphere were quite similar (Fig. 3). There was no significant difference in the qualitative composition of the total bacterial community with more than 1% abundance in bulk soil and rhizosphere of plants from both wild and cultivated areas. In bulk soil, Acidobacteria and Proteobacteria were relatively abundant in both wild (29.3% and 28.2%, respectively) and cultivated (33.9% and 33.4%, respectively) habitats. In rhizosphere, Proteobacteria and Acidobacteria were also abundant in wild (31.9% and 25.3%) and cultivated plants (38.7 and 30.5%, respectively). Overall, the composition and dominant phyla of the bacterial communities in the bulk soil and rhizosphere were similar between the wild and cultivated areas.

The most abundant bacteria in roots of wild U-SMN plants were Tenericutes (49.0%), followed by Proteobacteria (32.9%), whereas in cultivated plants, Proteobacteria was the most dominant (90.6%), followed by Actinobacteria and Bacteroidetes (4.4% and 2.4%, respectively). Most of the dominant phyla in wild plants were also present in the roots of cultivated plants. However, Tenericutes and Nitrospirae were not detected in the roots of the cultivated plants. The abundance of Tenericutes was relatively low in the bulk soil and rhizospheres of the wild and cultivated areas. Latescibacteria_WS3 present in the bulk soil and rhizosphere of both wild and cultivated plants was not detected in roots from either area (Fig. 3). Overall, the root endophytic bacterial community was distinct from that of the bulk soil and rhizosphere soil in both wild and cultivated habitats. In addition, the endophytes were significantly different between cultivated and wild plants at the phylum level, and the endophytes of cultivated plants were less diverse than those of endophytes in wild plants. These results indicate that cultivation simplified the bacterial community diversity. However, further studies are needed to understand the cause of this difference between the endophytes of wild and cultivated plants.

### Features of bacterial profiles at the genus and species level

**Figure 4:**
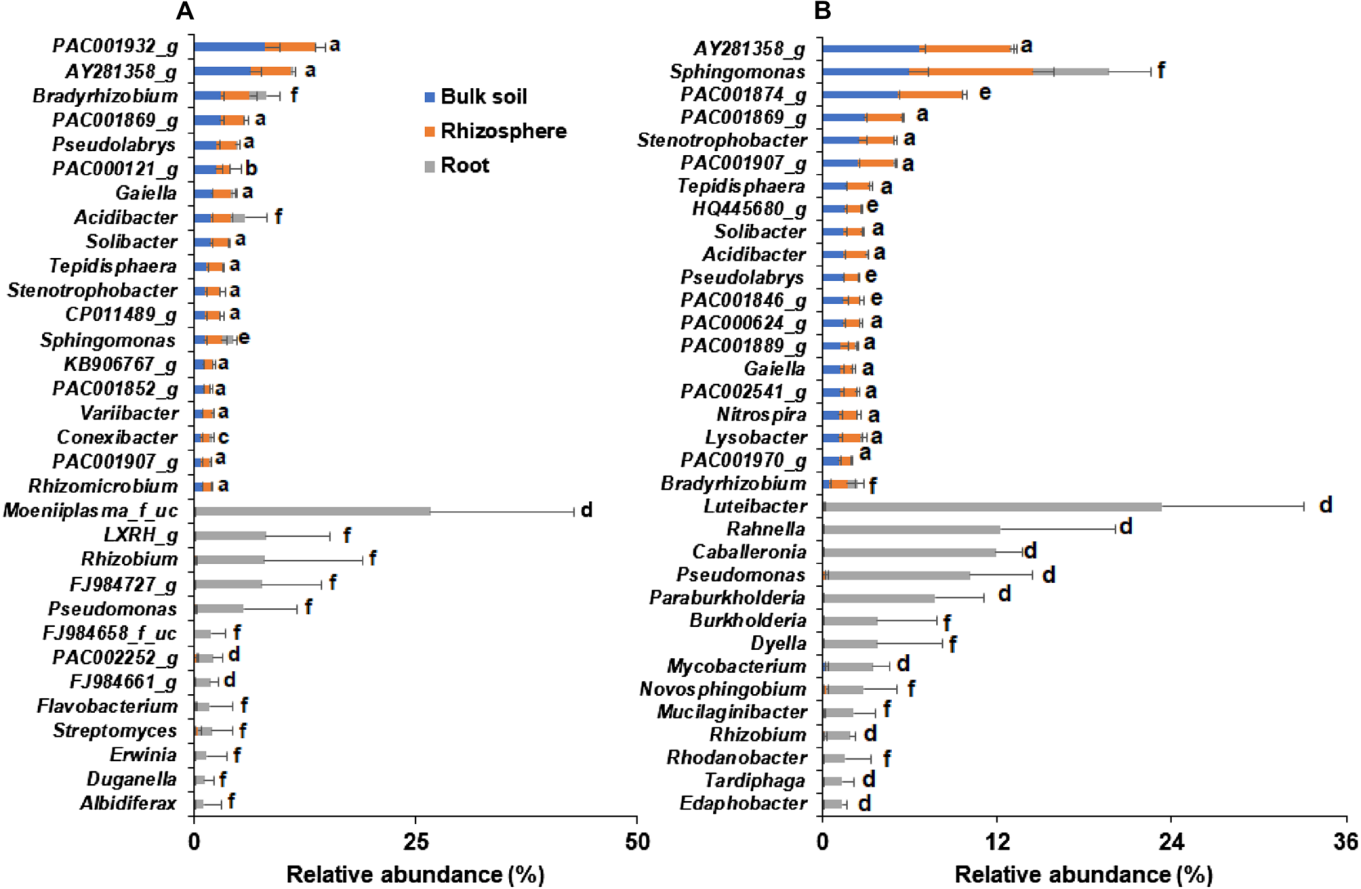
Comparison of relative abundance of bacteria at the genus level in bulk soil, rhizosphere, and root of Ulleung-sanmaneul plants. The bacterial genera with at least 1% relative abundance in each compartment of wild (**A**) and cultivated (**B**) habitats were selected for comparison. Data represent mean ± SD and letters in bars present significant difference at *P* = 0.05 as follows: (a) Bs = Rs > Ro, (b) Bs = Rs ≥ Ro, (c) Rs = Bs ≥ Ro, (d) Ro > Bs = Rs, (e) Bs > Rs > Ro and (f) Bs = Rs = Ro.

The bacterial composition at the genus level also showed differences in endophytic bacterial composition from those of the bulk soil and rhizosphere. In wild habitats, the relative abundance of genera belonging to Verrucomicrobia and Acidobacteria (PAC001932_g, AY281358_g, and PAC001869_g) was high in the bulk soil and rhizosphere, whereas *Moeniiplasma_*f_uc, PAC002252_g, and FJ984661_g was relatively abundant in roots (Fig. 4A). There was no significant difference in relative abundance between the compartments (Fig. 4A). Bacterial genera such as *Solibacter*, *Tepidisphaera*, *Stenotrophobacter*, and *Rhizomicrobium,* which were abundant in the bulk soil and rhizosphere, were not detected in the roots of the wild plants. In case of cultivated plants, the genera belonging to Verrucomicrobia and Acidobacteria were highly abundant in the bulk soil and rhizosphere, whereas *Luteibacter* (23.2%), *Rahnella* (12.2%)*, Caballeronia* (11.9%), and *Pseudomonas* (9.9%) were relatively abundant in roots (Fig. 4B). The bacterial genera *Stenotrophobacter*, *Pseudolabrys*, and *Nitrospira*, which were dominant in the bulk soil and rhizosphere, were not detected in the roots of cultivated plants. The genera *Paraburkholderia*, *Mycobacterium*, *Edaphobacter*, *Tardiphaga,* and *Rhizobium* were more abundant in the roots than in the bulk soil and rhizosphere of the cultivated plants.

Heat map analysis revealed similarities and differences in bacterial profiles between the rhizocompartments of wild and cultivated plants (Supplementary Fig. S2A,B). In the wild area, 138 genera were shared between bulk soil and rhizosphere (91% and 82% of the total population in the respective compartments) (Supplementary Fig. S2C), and in the cultivated area, 135 genera were shared between bulk soil and rhizosphere (89% and 84% of total population) (Supplementary Fig. S2D). In wild and cultivated areas, 13 and 4 genera, respectively, were common in all compartments, and 4 and 8 genera were common between the rhizosphere and root. *Sphingomonas* was abundant in the bulk soil and rhizosphere of the cultivated areas (Supplementary Fig. S2B), whereas PAC001932_g, belonging to Verrucomicrobia was abundant in the bulk soil and rhizosphere of wild plants (Supplementary Fig. S2A).

We further compared the endogenous bacterial community structure in the roots of wild and cultivated plants (Supplementary Fig. S3A,B). Among the genera showing > 1% relative abundance, *Luteibacter* (23.2%)*, Rahnella* (12.2%)*, Caballeronia* (11.9%)*, Paraburkholderia* (7.7%)*,* and *Burkholderia* (3.8%) were dominant in the roots of cultivated plants, whereas genera belonging to Tenericutes like *Moeniiplasma_*f_uc (26.7%), LXRH_g (8.1%), and FJ984727_g (7.6%), and others, such as *Bradyrhizobium* (2.0%)*, Acidibacter* (1.7%), and *Flavobacterium* (1.6%), were abundant in roots of wild plants. The compositions of the top 15 genera in the roots of wild and cultivated plants were different, except for three common genera, namely *Pseudomonas, Rhizobium,* and *Sphingomonas*. Overall, the results indicated that the bacterial compositions of the bulk soil and rhizosphere at the genus level were similar between the wild and cultivated areas. The bacterial structure of the roots was similar, with different relative compositions between habitats.

**Figure 5:**
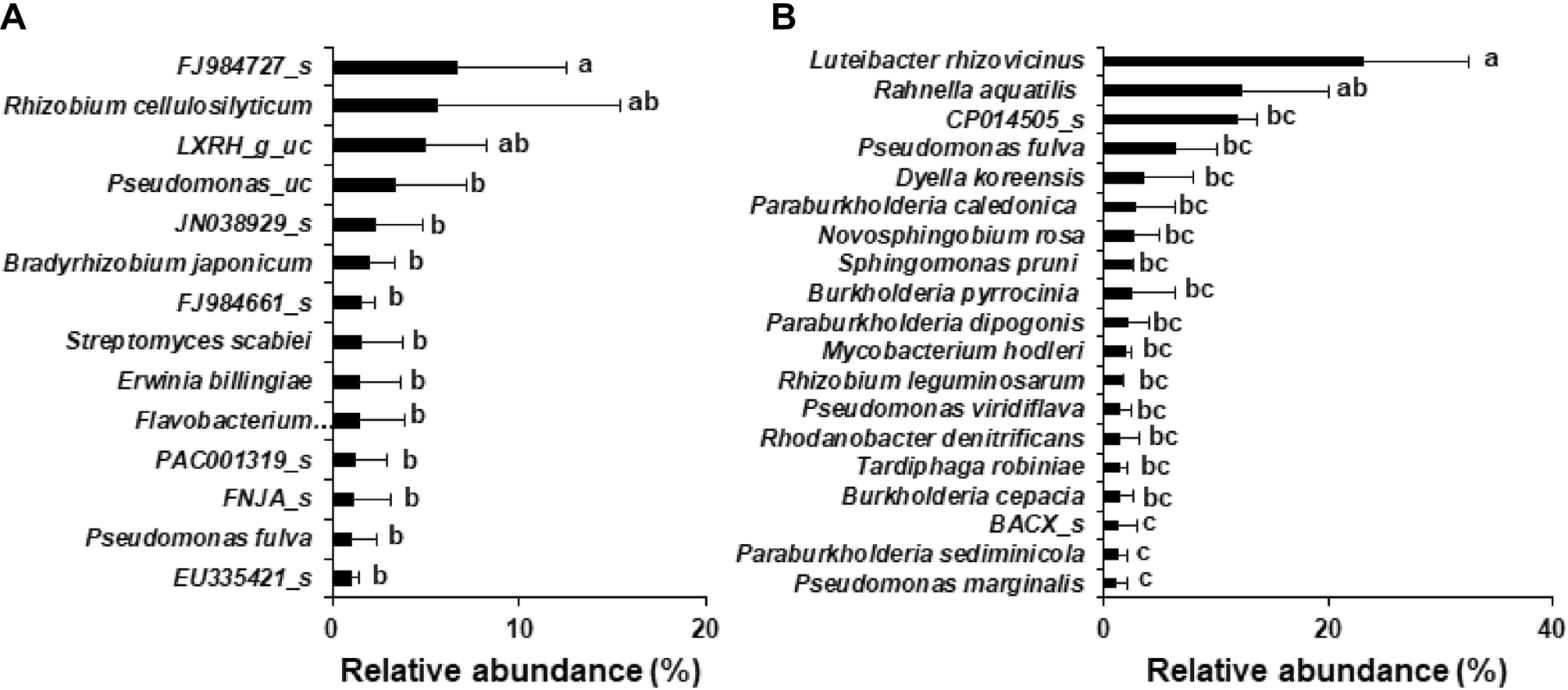
Relative abundance of bacterial species within the roots of Ulleung-sanmaneul plants. The bacterial species with relative abundance > 1% in roots were compared between wild (**A**) and cultivated (**B**) habitats. The data are represented as mean ± standard deviation and bars with the same letter do not differ significantly at *P* = 0.05.

At the species level, *Bradyrhizobium japonicum* was the most abundant species in bulk soil and rhizosphere (3.0% and 3.1%, respectively) of wild habitat, followed by PAC001932_g_uc (2.2% and 1.5%, respectively), which belong to Proteobacteria (Supplementary Fig. S4A). In roots of wild plants, 14 species such as FJ984727_s (6.6%), LXRH_g_uc (5.0%), JN038929_s (2.3%), and FJ984661_s (1.6%) that belong to Tenericutes; *Rhizobium cellulosilyticum* (5.6%), *Pseudomonas* sp. (3.3%), and *Bradyrhizobium japonicum* (2.0%), showed more than 1% relative abundance with similar proportion (Fig. 5A). Among the bacterial species with relative abundance > 0.1%, *Sphingomonas pruni* and *Edaphobacter modestus* were shared by all compartments of wild plants, and four species such as *Streptomyces scabei*, *Luteibacter rhizovicinus*, *Herbiconiux ginseng*, and *Leifsonia poae* were identified both in rhizospheres and roots of wild plants*.* The abundant species in the bulk soil and rhizosphere of cultivated habitat were AY281358_g_uc belonging to Acidobacteria (1.8% and 2.0%, respectively), *Sphingomonas lutea* (1.3% and 1.9%, respectively), and *Sphingomonas sediminicola* (1.1% and 1.8%, respectively) (Supplementary Fig. S4B). In the roots of cultivated plants, 19 species such as *Luteibacter rhizovicinus* (23.0%), *Rahnella aquatilis* (12.2%), and species belonging to *Pseudomonas, Paraburkholderia* and *Sphingomonas* exhibited more than 1% relative abundance (Fig. 5B). The species in the roots of cultivated plants were distinct from those in the rhizosphere, except for *Luteibacter rhizovicinus*, and *Sphingomonas pruni.*

Among the top 25 endophytes, those common in the roots of wild and cultivated plants were *Pseudomonas fulva* (1.0% and 6.4%, respectively), *B. japonicum* (2.0% and 0.7%, respectively), and *Tardiphaga robiniae* (0.7% and 1.4%, respectively) (Supplementary Table S4).

### Comparison of total culturable bacteria

The total number of culturable bacteria was compared between the compartments of wild and cultivated U-SMN plants. In the wild habitat, there was no significant difference in the total number of culturable bacteria between bulk soil, rhizosphere, and roots, whereas the population was slightly lower in the roots. In the cultivated areas, the total bacterial population was higher in the bulk soil and rhizosphere than in the roots (Supplementary Fig. S5). The results indicated that culturable bacterial microbiota was more abundant in the surrounding soils than in the roots, which corresponds to the results of 16S rDNA profiling.

### Growth promotion and salt stress alleviation of Arabidopsis by co-inoculation of JBCE485 and JBCE486

Among the 183 strains screened, JBCE485 and JBCE486, which were isolated from bulk soil of U-SMN in wild habitats, significantly increased the growth of Arabidopsis and were identified as *Pseudoxanthomonas* sp. (NCBI Acc No. OM780199) and *Variovorax paradoxus* (Acc No. OM780219), respectively, as determined by 16S rDNA sequencing. The growth-promoting assay using various cell concentrations of both strains indicated that 1 × 10^7^ CFU/mL is the optimum cell concentration for the treatment of plant seeds in view of the growth-promoting performance and dilution factor for mass application (Supplementary Figs. S6, S7).

**Figure 6:**
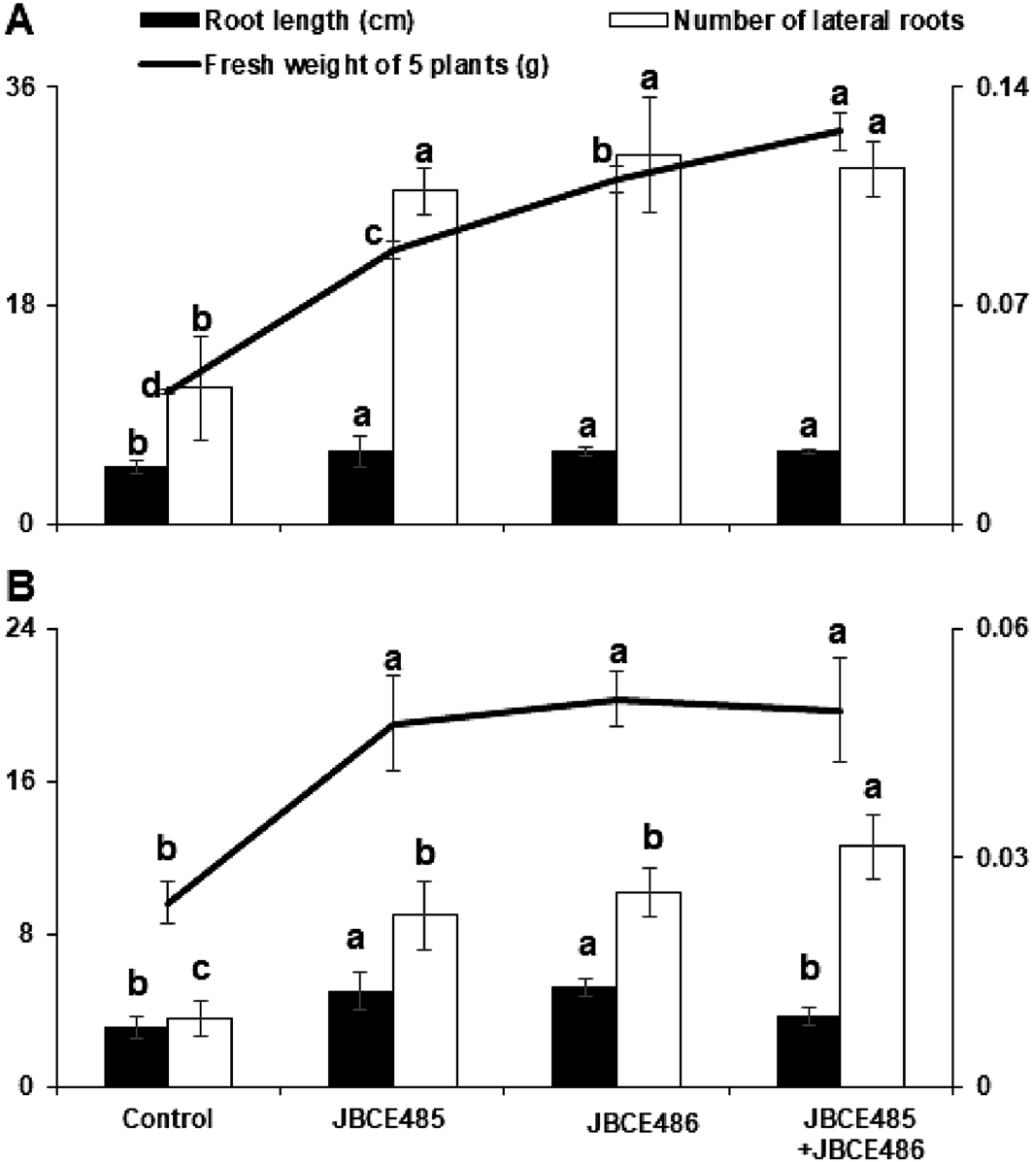
Growth promotion and salt stress alleviation of Arabidopsis by treatment with *Pseudoxanthomonas* sp. JBCE485 and *Variovorax paradoxus* JBCE486. Seeds of *Arabidopsis thaliana* Col-0 were treated with 1 × 10^7^ CFU/mL cells of individual or combined strains of JBCE485 and JBCE486. Seeds were grown on ½ MS medium without (**A**) or with (**B**) 100 mM NaCl. The left Y-axis indicate root length (cm) and number of lateral roots, and right Y-axis indicate fresh weight of five plants (g). The data are represented as mean ± standard deviation and bars with the same letter do not differ significantly at *P* = 0.05.

Microbial strains can be selected and combined to form synthetic microbiomes that enhance the performance of the rhizosphere. To use both strains as co-inoculants for successful colonization and growth-promoting activity, we assessed their compatibility. The strains JBCE485 and JBCE486 did not inhibit each other’s growth in cross-culture and dual inoculation assays, indicating that both strains are compatible for simultaneous use (Supplementary Fig. S8). Under no salt stress conditions, the treatment with individual and combined strains increased root length, number of lateral roots, and fresh weight of plants compared to the untreated control (Fig. 6A, Supplementary Fig. S9A). Intriguingly, the phenotypes of Arabidopsis differed between the individual and combined treatments. Roots were long and thin after treatment with JBCE485, and the main and lateral roots were thickened by treatment with JBCE486 compared to the JBCE485 treatment, while the number of lateral roots was similar between individual and combined treatments. The fresh weight was significantly increased by treatment with the combined strains compared with the individual treatments (Fig. 6A). Under salt stress conditions, individual and combined bacterial treatments increased root length compared with the untreated control. Treatment with JBCE485 produced thin and long roots, whereas JBCE486 produced thick and long roots with more lateral hairs, which was similar to the phenotypes under no salt stress conditions (Supplementary Fig. S9B). The number of lateral roots was increased by the combined treatment compared to the individual treatments, while the combination treatment showed similar root length to that of the control (Fig. 6B). The increase in root length or lateral root number by treatment with bacteria contributed to the overall increase in fresh weight compared to the untreated control. Overall, the results indicated that co-inoculation of bacterial strains promoted Arabidopsis growth with different phenotypes compared to individual treatments under normal and salt stress conditions.

### Growth promotion and salt stress alleviation of chive by JBCE485 and JBCE486

**Figure 7:**
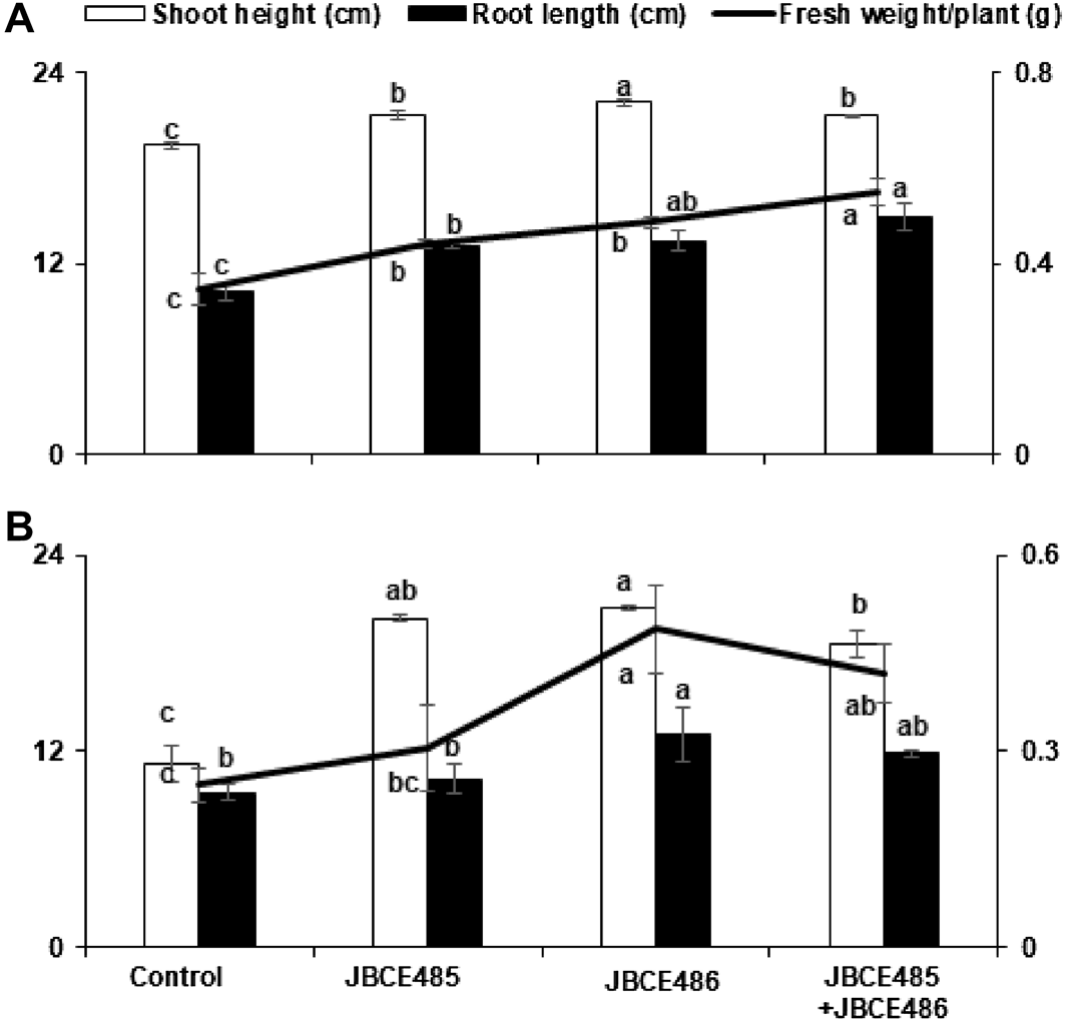
The effect of *Pseudoxanthomonas* sp. JBCE485 and *Variovorax paradoxus* JBCE486 on the growth of chive under salt stress conditions. Seeds of chive were treated with 1 × 10^7^ CFU/mL cells of each bacterium JBCE485 and JBCE486, and their mixture. Seeds were grown on horticultural nursery soil soaked with tap water (**A**) with or (**B**) without 175 mM NaCl (soil/water, 2:1 v/v). The left Y-axis indicates shoot height (cm) and root length (cm), and the right Y-axis indicates fresh weight of plants (g). Growth was recorded at 4 and 6 weeks after cultivation in soil without or with salt, respectively. The data are represented as mean ± standard deviation and bars with the same letter do not differ significantly at *P* = 0.05.

**Figure 8:**
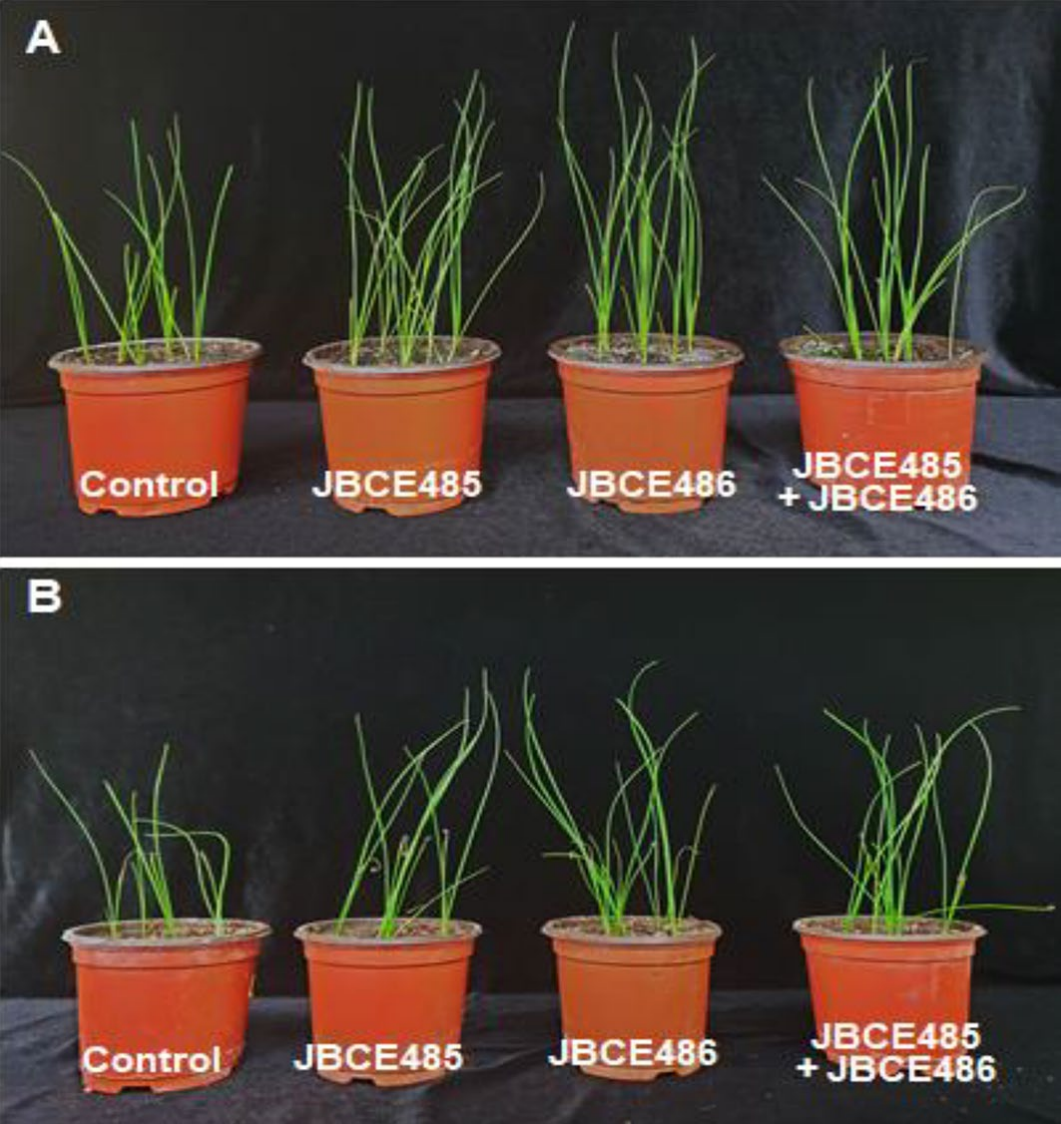
Stress alleviation of chive by treatment with *Pseudoxanthomonas* sp. JBCE485 and *Variovorax paradoxus* JBCE486. Chive seeds were surface sterilized and treated with 1 × 10^7^ CFU/mL cells of individual and combination of JBCE485 and JBCE486. Seeds were grown on normal horticultural soil soaked with tap water (**A**) with or (**B**) without 175 mM NaCl. Representative photos of plants were taken after 4 weeks and 6 weeks of treatment in soil without or with salt, respectively.

Chives that belong to *Allium* crops, such as U-SMN, were used to assess the growth promotion and salt stress alleviation effects of co-inoculation. Under soil conditions without salt stress, the treatment of chive seeds with individual and combined cells of JBCE485 and JBCE486 prompted germination speed and seedling growth compared to the untreated control (Figs. 7A, 8A). The shoot height of plants treated with combined strains was similar to that of plants treated with JBCE485, whereas root length was similar to that of plants treated with JBCE486. The fresh weight of chive plants was significantly increased by co-inoculation compared with the individual treatments (Fig. 7A). Under salt stress conditions, germination of chive seeds was delayed, and growth was retarded with yellowish leaves and dried tips compared to normal soil. The individual and combined treatments of JBCE485 and JBCE486 resulted in greener and healthier plants than the control plants (Figs. 7B, 8B). In addition, treatment with JBCE486 and the combined strains increased the fresh weight of the plants (Fig. 7B). Overall, our results indicate that treatment with combined bacterial strains enhanced chive growth and induced resistance against salt stress with distinct beneficial effects.

### Traits of JBCE485 and JBCE486 for plant growth promotion

To understand the mode of action for plant growth promotion, nutrient mobilization and phytohormone production activities were compared between each strain and the co-inoculation. Both the JBCE485 and JBCE486 strains produced siderophores, and the size of the orange halo was similar between the individual and combination of strains (Supplementary Table S5). JBCE485 produced protease and there was no significant difference in the size of the clear zone between co-inoculation and JBCE485 alone. The indole acetic acid (IAA) production by combined strains (3.75 µg/mL) was significantly increased compared to the individual JBCE485 (3.16 µg/mL) and JBCE485 (1.79 µg/mL) strains (Supplementary Table S5). Neither of the strains solubilized phosphate nor produced cytokinins.

### Growth patterns of Arabidopsis under phytohormone biosynthesis suppression conditions

Individual and combined treatments with JBCE485 and JBCE486 promoted Arabidopsis and chive growth with different effects and phenotypes. To understand the phytohormones responsible for the changes in phenotypes by bacterial treatment, the growth patterns of Arabidopsis Col-0 were observed under the application of various hormone biosynthesis inhibitors. The growth of Arabidopsis is significantly reduced by treatment with hormone inhibitors. The growth of Arabidopsis by treatment with individual and combined strains under hormone inhibitors was similarly suppressed as in untreated Col-0, except for AOA (inhibitor of auxin and ethylene). Under AOA treatment, JBCE485 enhanced the growth of Arabidopsis by increasing root length and the number of lateral roots, and JBCE486 increased root length and the number of root hairs (Supplementary Fig. S10). Co-inoculation increased Arabidopsis growth, phenotype was observed to be in between the phenotypes of JBCE486 and JBCE485. In addition, JBCE485 and the combined treatment significantly increased the growth of the Arabidopsis *eir1* mutant compared to the untreated control (Supplementary Fig. S11). Taken together, these results indicate that treatment with bacterial strains overcame the suppression of auxin biosynthesis in plants, which promoted the growth of Arabidopsis.

### Population dynamics and localization of each bacterial strain in individual and co-inoculated rhizosphere

The application of multiple strains can enhance the ability of beneficial microorganisms to colonize and maintain stable communities in the treated rhizosphere. When each strain was individually inoculated into Arabidopsis seed, the population of JBCE486 and JBCE485 was maintained at a similar level in the plant roots at 7 days (1.7 × 10^5^ and 1.5 × 10^5^ CFU/g, respectively) and 14 days (1.3 × 10^5^ and 1.2 × 10^5^ CFU/g, respectively) after treatment (Supplementary Fig. S12). In the combined treatment, the population of JBCE486 was higher (1 × 10^5^ CFU/g roots) than JBCE485 (0.7 × 10^5^ CFU/g) at 7 days after treatment, and the population of JBCE486 was maintained higher than that of JBCE485 until 14 days (Supplementary Fig. S12). The total number of bacterial cells (JBCE485 + JBCE486) was similar to the total number of JBCE486 or JBCE485 cells after 7 days of treatment. However, the total number of bacteria in the combination treatment was lower than the population of each bacterial treatment 14 days after incubation (Supplementary Fig. S12). These results indicate that there is no difference in the colonization efficiency of Arabidopsis roots between JBCE485 and JBCE486. However, when applied together, JBCE486 cells survived more efficiently in Arabidopsis roots than JBCE485.

**Figure 9:**
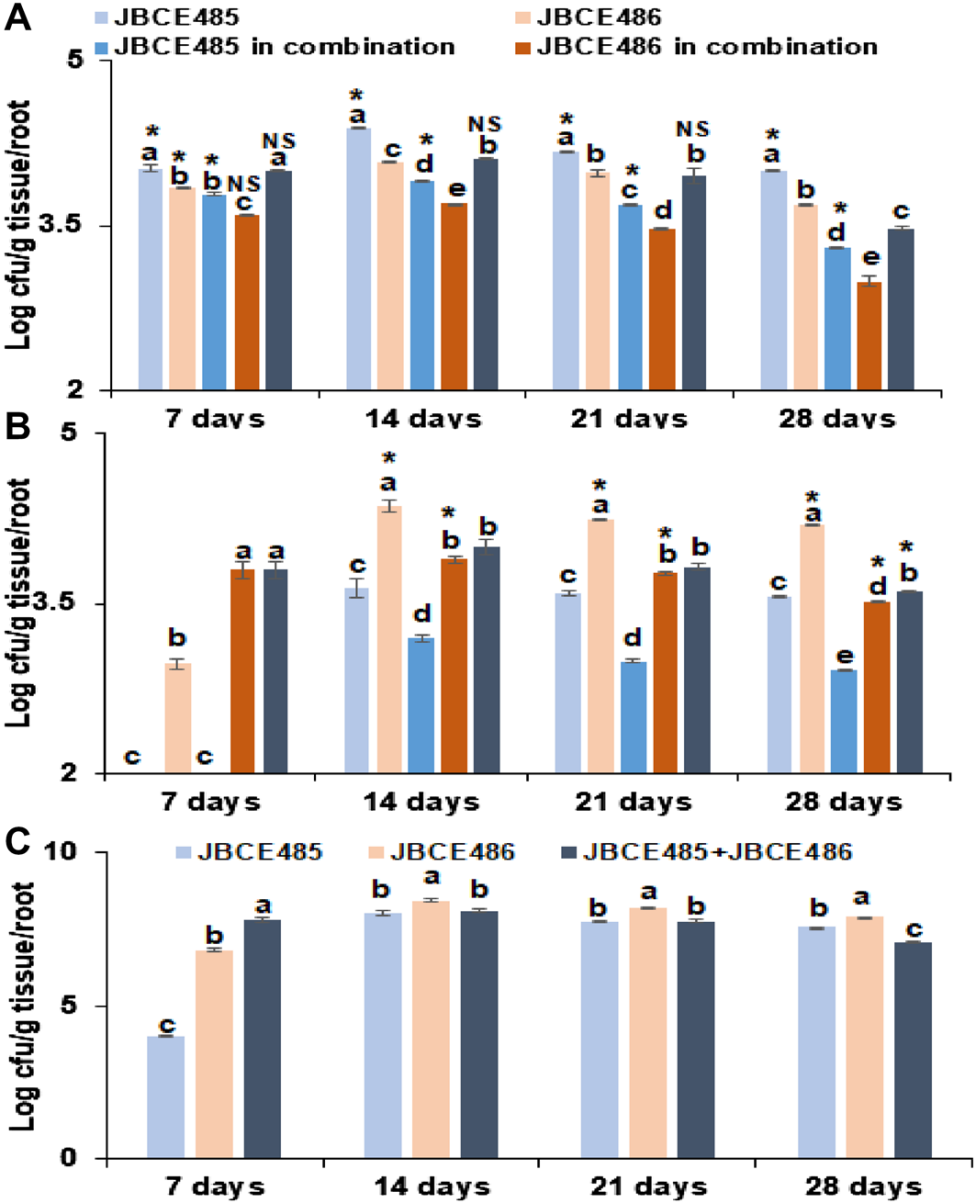
Population dynamics of *Pseudoxanthomonas* sp. JBCE485 and *Variovorax paradoxus* JBCE486 cells on chive roots. Seeds were treated with individual and combined cell suspensions (1 × 10^7^ CFU/mL) of JBCE485 and JBCE486 and sown in horticultural soil. (**A**) Each bacterial population on the root surface at 7, 14, 21, and 28 days after treatment. All tissues below soil surface at 7 days after growth and roots at 14, 21, and 28 days after growth were collected and cells per g tissues were estimated by serial dilution plating on LB medium containing rifampicin (25 µg/mL) and tetracycline (25 µg/mL) for JBCE485 and JBCE486, respectively. (**B**) Each bacterial population inside of roots. Surface sterilized roots were macerated in buffer and plated on LB media. (**C**) Total population of bacteria residing on the surface and inside of roots. The experiment was replicated two times with 15 seedlings per replicate. The data are represented as the standard deviation of 5 independent biological samples. Bars with the same letter(s) do not differ significantly at *P* = 0.05 at each timepoint for respective compartments. *Indicates significant difference at *P* = 0.05 of a treatment with respect to niche; *NS* not significant.

When chive seeds were treated with the bacterial strain individually, both the JBCE485 and JBCE486 strains colonized and were well maintained on the chive roots for 28 days. The population of JBCE485 on the root surface of the chive was higher than that of JBCE486 in both the individual and combined treatments (Fig. 9A). The total bacterial cells in the combined treatment were similar to that in treatment with JBCE485 alone in the first 7 days (1 × 10^4^ CFU/g), but the total population of the combined treatment decreased and was maintained lower than the population treated with JBCE485 alone, whereas it was similar to that of treatment with JBCE486 alone until 21 days (Fig. 9A). However, the combined population was lower than the individual treatment with either JBCE485 or JBCE486 at 28 days. When we investigated the population of each bacterial strain inside the chive roots, JBCE486 maintained a higher population than JBCE485 both in the individual and combined treatments (Fig. 9B). In the combination treatment, the total population inside the roots was less than that of JBCE486 alone, but higher than that of JBCE485 alone (Fig. 9B). The population levels of each strain in the combined treatment were lower than those in individual treatments. The total population of bacteria on the surface and inside of roots by combination treatment was higher than that of individual treatment at 7 days, whereas later the total population was maintained similar to that of JBCE485 alone (Fig. 9C). Taken together, JBCE485 colonized the root surface, while JBCE486 resided as an endophyte in the roots of chive. The results indicate that co-inoculation of both strains increase successful colonization inside and on the surface of roots without niche competition.

## Discussion

The plant microbiome has been recognized as an important determinant of plant growth and fitness and has been extensively studied to enhance the productivity, stress resistance, and health of plants^24,25^. However, the structures of the rhizosphere and endogenous microbiome of endemic plants in wild habitat ecosystems and changes in microbial community structures by domestication remain underexplored. In this study, we analyzed the rhizosphere and endophytic bacterial community of U-SMN plants, which are endemic to Ulleung Island, Korea, and compared them with those of cultivated U-SMN plants.

Our results indicated that bacterial diversity and taxonomic structure were higher in soils surrounding U-SMN plants than in those of wild and cultivated areas. Plants select and shape rhizosphere microbes by actively secreting compounds that stimulate or repress microbial members. Similar microbial communities are maintained even in different soils by plant species^26^. In this study, the bacterial community structures of the bulk soil and rhizosphere in both wild and cultivated habitats were similar, despite significant differences in soil properties. This might be due to host selection processes that moderate the impact of agricultural management in maintaining similar rhizosphere communities^27^. The buffering effect of host plants neutralized the alterations of domestication and might induce the co-occurrence of microbes irrespective of habitat and soil type^28^. These results indicate that host plant selectivity is more influential than environmental factors. However, the overall endophytic bacteria of cultivated U-SMN plants were less diverse than those of endophytes in wild plants. Detailed analysis of the bacterial community at the genus and species levels revealed that the endophytes of roots differed in relative composition rather than taxonomic structure between habitats. The domestication of plants and cultivation practices has been known to affect soil properties and also the root exudates of plants, which, in turn, influences the soil microbiome^12,29–31^. Hassani et al.^28^ suggested that the bacterial and fungal community patterns in wheat are distinct between wild and domesticated species. As observed in our study, the difference in concentration of ions in cultivated soil might influence the growth of plants thereby affecting the harboring endophytic bacterial community. Moreover, domestication and continuous breeding influence vertically transferred bacteria more than soil-derived microbiota^32^. Increased nitrogen application in Welsh onion reduced the relative abundance of Acidobacteria, Verrucomicrobia, and Sphingobacteria^33^ which is similar to our results. The culturable bacteria in the bulk soil, rhizosphere, and roots of both wild and cultivated U-SMN plants also indicated that total bacteria in the roots of cultivated plants were lower than those in the surrounding soils. The current and previous results indicate that cultivation simplifies the bacterial diversity. Further studies are needed to understand the cause of this difference between the endophytes of wild and cultivated plants.

In this study, we investigated the bacterial structure in the bulbs of U-SMN plants to analyze consistent endophytes which might belong to the core microbiome. Although the results were not included in the main analysis because of low reads in one or two biological replicates, we used 16S rDNA sequencing data to identify the presence or absence of endophytic bacteria at the species level. Fourteen species, including *Erwinia billingae*, *Pantoea agglomerans*, and *Pseudomonas fulva* were common endophytes in the roots and bulbs of wild plants. In cultivated plants, 14 species, including *Luteibacter rhizovicinus*, *Rahnella aquatilis,* and species belonging to *Paraburkholderia* and *Pseudomonas* were shared between roots and bulbs. Among these species, *Luteibacter rhizovicinus*, *Pseudomonas fulva*, *Sphingomonas pruni,* and CP014505_s (belonging to *Caballeronia*) were detected as endophytes in the bulb and root of both wild and cultivated plants, indicating that these species might belong to the core bacterial species of U-SMN plants, which co-occur irrespective of soil type and habitat alteration. *Pseudomonas* has been identified as a plant-specific signature for Himalayan onion (*A. wallichii* Kunth)^34^, *Sphingomonas* sp. as a beneficial endophyte in *Allium*^35^, and *Luteibacter* as a prominent leaf endophyte that helps to adapt host plants in karst regions^36^. It is expected that *Pseudomonas, Sphingomonas* and *Luteibacter* might be a part of the core microbiome of U-SMN. The influence on growth and adaptation of U-SMN requires further analysis.

The JBCE485 and JBCE486 strains, which were isolated from the bulk soil of U-SMN plants, were selected for the promotion of growth and salt stress resistance in Arabidopsis and chive. *Pseudoxanthomonas* sp. and *Variovorax paradoxus* have already been reported to promote growth of plants^37–39^. The application of beneficial microbial agents has mainly focused on the selection of an effective, single microbial species. However, in natural habitats, the beneficial effects of plant-associated microbes result from the synergistic interaction of multiple microbes in the niche. Combination of JBCE485 and JBCE486 improved the growth promoting performance compared to individual treatments with phenotypic alterations which were more prominent in roots. There are multiple reports emphasizing the application of microbial consortia to improve efficacy and consistency since mixed strains can adapt to a broader range of environmental conditions^20,21^. The combination of growth-promoting strains *Bacillus subtilis* GB03 and *B. amyloliquefaciens* IN937a provided a higher control efficacy against *Fusarium oxysporum* f. sp*. radicis-lycopersici* on tomato than the individual strain, indicating combined applications have higher efficacy in terms of growth promotion as well as biotic and abiotic stress alleviation^40–42^. Our studies under salt stress condition also indicated that combination of strains provide increased beneficial effect on growth of plants than individual bacterial application. The selected beneficial strains can be further studied for improvement of U-SMN, both for application in wild habitat and implementation in cultivated areas for betterment of the crop growth and adaptation.

Beneficial microbes promote plant growth or induce resistance to biotic and abiotic stresses through several mechanisms, such as nutrient mobilization, phytohormone production, competition for space and nutrients, and antibiosis^43^. Moreover, the traits of individual microbes can be combined to enhance their beneficial effects^44^ and the traits in the community cannot be predicted from individual members. In this study, JBCE485 and JBCE486 were compatible in the culture media without inhibiting each other, indicating that both strains could be used together for synergistic effects. The IAA producing *V. paradoxus* strain 5C-2 reported to improve root growth^39^. The analysis of our study indicated that the quantitative production of IAA was higher in the co-inoculated culture medium than in individual cultures. The difference in growth patterns and phenotypes of Arabidopsis and chive after treatment with individual and co-inoculation of both strains might be due to the variation in concentration of auxin in co-inoculated plant roots^45,46^. Therefore, further studies are required to understand the mechanisms of action for growth promotion and stress resistance by JBCE485 and JBCE486.

Co-inoculation of bacterial strains could increase the performance of each strain because different types of bacteria might interact synergistically to enhance beneficial effects. However, to exert the beneficial effects, the inoculated strains need to sufficiently colonize the treated niches. Our results indicate that whether individually or in combination, JBCE485 and JBCE486 sufficiently colonize plant roots to promote growth and induce salt stress alleviation. Interestingly, JBCE485 mostly colonized the root surface of chive plants, whereas JBCE486 predominantly resided inside the roots. This indicates the co-survival of respective strains in different root compartments, which thereby exerts beneficial effects on plants without niche competition. In addition, co-survival in different root compartments may provide stable colonization under changeable environmental conditions.

Overall, the results of this study indicate that the selectivity of U-SMN plants is more influential than that of soils in formulating endogenous bacteria. The domestication of the plant for commercial cultivation simplified bacterial diversity. To the best of our knowledge, this is the first report of bacterial community structures associated with the rhizospheres and roots of U-SMN plants. The selected beneficial bacterial strains, JBCE485 and JBCE486, promoted growth and induced salt-stress resistance in Arabidopsis and chive plants. Co-inoculation with both JBCE485 and JBCE486 improved the performance of plant growth-promoting activities probably with an increase in IAA and successful colonization. The information obtained from this study will contribute to understand the U-SMN associated bacterial community which may further help us to identify and develop important species necessary for growth and adaptation of U-SMN. The differences of endophyte in wild and cultivated U-SMN plants suggested the necessity of manipulating endogenous bacteria for the plant growth. We will further study to develop synthetic consortia to improve the potentials of beneficial activities. In addition, we expect that the use of the selected strains will increase targeted metabolites in Allium plants.

## Materials and methods

### Sampling of plants and sample processing

Sampling was conducted during the growing season (June–July 2020) at two sites: The wild habitats located in the mountain hillside of Jeodong-ri, Ulleung-eup Ulleung-gun, Gyeongsangbuk-do, South Korea, with the permission of authorities of Baekdudaegan National Arboretum, Korea. The cultivation areas located at the Medicinal Herb Experiment Station, Jinan-gun, Jeollabuk-do Agricultural Research and Extension Services, Korea. From the plant collection areas, two individual Ulleung-sanmaneul (*Allium ulleungense* H. J. Choi & N. Friesen; U-SMN) plants (spaced 30 cm apart) were randomly dug out with intact roots along with soil from 5 cm outside of the root zone and combined to form one biological replicate and stored in sterile plastic bags at 4 °C. The samples were brought to the laboratory and separated into bulk soil, rhizosphere soil, and endosphere (root) samples^47^. Bulk soil was collected from the root system by gentle shaking, and the detached particles were then sieved through a 2-mm sieve. Subsequently, the roots were vigorously shaken to remove adherent soil until only firmly attached soil remained. The attached soil was collected using sterilized brushes as the rhizosphere soil sample. The endosphere samples from roots were washed with tap water and surface disinfected using 70% (v/v) ethanol for 1 min, followed by 3% (v/v) sodium hypochlorite solution for 3 min, and five rinses of sterile water. The final rinse (100 μL) was cultivated on Luria Bertani (LB) medium plates and examined for bacterial growth after incubation at 30 °C for 48 h to confirm that surface sterilization was successful. Bulb samples collected from wild and cultivated plants were also processed following the same steps as the root sample. Three biological samples from the bulk soil, rhizosphere, and endosphere were analyzed. All plant experiments were performed in accordance to relevant regulations and guidelines.

### DNA isolation and 16S rDNA sequencing

DNA was extracted using the GeneAllExgene™ Soil DNA isolation kit (GeneAll, South Korea) following the manufacturer’s instructions and analyzed for purity using BioTek, Epoch™ Spectrometer (USA), and 1% agarose gel electrophoresis. Bacterial libraries were generated by PCR to amplify the V3–V4 region of the 16S rRNA gene using universal primers 341F (5′-CCTACGGGNGGCWGCAG-3′) and 805R (5′-GACTACHVGGGTATCTAATCC-3′), which contain Nextera consensus and adaptor sequences at the forward (5′-TCGTCGGCAGCGTC-AGATGTGTATAAGAGACAG-target sequence-3′) and reverse (5′-GTCTCGTGGGCTCGG-AGATGTGTATAAGAGACAG-target sequence-3′) primers^48^. The PCR products were cleaned up, and further amplification was performed using primers containing Illumina dual indices and sequencing adapters: forward index i5 (5′-AATGATACGGCGACCACCGAGATCTACAC-55555555-TCGTCGGCAGCGTC-3′) and reverse i7 (5′-CAAGCAGAAGACGGCATACGAGAT-7777777-GTCTCGTGGGCTCGG-3′). The PCR conditions were as follows: initial denaturation at 94 °C for 3 min, followed by 25 cycles of denaturation at 94 °C for 30 s, annealing at 55 °C for 30 s, extension at 72 °C for 30 s, and a final extension at 72 °C for 5 min. The Quant-iT PicoGreen dsDNA Assay Kit (Invitrogen, USA) was used to quantify the PCR products, and their quality was checked using an Agilent Bioanalyzer 2100 system. Purified amplicon libraries were pooled and sequenced at ChunLab Inc. (South Korea) on an Illumina MiSeq system using the MiSeq Reagent Kit v2 (Illumina Inc., USA).

### Sequence data processing and analysis

EzBioCloud (https://www.ezbiocloud.net/), an in-house pipeline developed by ChunLab Inc. (Seoul, South Korea)^47,49^ was used to analyze the sequenced data. The pipeline is used for quality control, merging of forward and reverse paired-end reads, sequence processing and taxonomic classification, and diversity analysis of OTUs. The raw sequencing reads were filtered out as low-quality (average quality value < 25) reads using Trimmomatic v0.32. Paired-end sequences were merged using PandaSeq^50^ and the EzBioCloud program was used to trim the primers at a similarity cut-off of 0.8. DUDE-Seq software^51^ denoised the sequence errors via the EzBioCloud 16S rRNA database, which manually removes chimeric sequences through UCHIME^52^. The EzBioCloud database was searched using the USEARCH program^53^ for implementation of taxonomic assignment, and sequence similarity was calculated via pairwise alignment. Matching of the query sequences to the reference sequence at 3% sequence dissimilarity was considered to be identified at the species level. The cluster database at high identity with tolerance (CD-HIT) and UCLUST tools with 97% similarity^54^ clustered the sequences that did not match to the database. The final set of OTUs was a combination of species identified in the EzBioCloud database and OTUs obtained using CD-HIT and UCLUST. The following were the cutoff values for other reference sequences: (x = similarity), genus (97% > x ≥ 94.5%), family (94.5% > x ≥ 86.5%), order (86.5% > x ≥ 82%), class (82% > x ≥ 78.5%), and phylum (78.5% > x ≥ 75%)^55^.

The bacterial diversities were analyzed and compared using CL community™ v3.43 (ChunLab, Seoul, South Korea)^47,56^. Rarefaction curves, alpha diversity analysis, abundance-based coverage estimator (ACE), Chao1, Shannon, Simpson, and phylogenetic diversity indices were calculated. The beta diversity, including principal coordinate analysis (PCoA), was analyzed based on the Bray–Curtis dissimilarity index^57^ at the genus and species level. Differences in alpha diversity for number of OTUs, richness and diversity were analyzed between the sampling sites. The difference in taxonomic composition between compartments (bulk soil, rhizosphere, and root) were also compared at the phylum to species level. The least significant difference (LSD) test at *P* = 0.05 was used to determine the relative differences of bacterial population between bulk soil, rhizosphere and roots in respective habitats. The differences between bulk soil and rhizosphere at species level were determined using the *t*-test at *P* = 0.05.

### Culture dependent microbial populations and identification of strains

Total bacterial populations were determined by serial dilution and plating on 1/10th LB agar and 1/10th TSA agar media with 50 µg/mL cycloheximide. The plates (two duplicate plates for each subsample/dilution combination) were incubated at 30 °C for 3 days prior to the enumeration of viable colonies. Random bacterial colonies with distinctive colors and shapes were picked and transferred individually to 1.5 mL tubes containing LB medium with 15% (v/v) glycerol. The tubes were incubated for 24 h at 30 °C and stored at − 80 °C for further analysis. Strains were identified by sequencing the 16S rDNA using primers 27F and 1492R and aligned with the NCBI 16S rDNA sequences.

### Soil physicochemical analyses

Bulk soil samples collected from each habitat were air-dried in the shade at room temperature and passed through a 2-mm sieve. Soil samples were mixed with distilled water (DW) (1:5 w/v) for 30 min at 200 rpm. The pH and electrical conductivity (EC) of the soils were determined using a pH meter (HI 9124, Hanna Instrument, USA) and an EC meter (HI9033, Hanna), respectively. The exchangeable cation content was determined by mixing 5 g of soil with 50 mL of ammonium acetate (1 N, pH 7.0) solution for 30 min at 200 °C and filtering to remove soil particles. Inductively coupled plasma optical emission spectrometry (ICP-OES; 5800 ICP-OES, Agilent, USA) was used to analyze the exchangeable Ca^2+^, K^+^, Na^+^ and Mg^2+^ contents^58^.

### Arabidopsis growth promotion assay

The bacterial strains isolated from U-SMN and stored at − 80 °C were revived in LB agar plates and incubated at 30 °C for 48 h. The bacterial cells were harvested from the plates in sterile DW (ca. 1 × 10^8^ CFU/mL) supplemented with 0.2% sterilized carboxymethyl cellulose (CMC). *Arabidopsis thaliana* Columbia-0 (Col-0) seeds were surface sterilized and soaked in each bacterial suspension. The suspensions were incubated at room temperature (approximately 25 °C) in a rotary shaker at 150 rpm for 30 min to facilitate bacterial cell attachment to the seed coat. The seeds were placed in Petri dishes with sterilized filter paper to remove excess moisture. The seeds soaked in sterile DW amended with 0.2% CMC served as controls. Bacterized seeds (5 seeds/plate) were placed onto Petri dishes (90 × 15 mm) containing half-strength Murashige and Skoog (½ MS) medium supplemented with 1.5% sucrose and 0.8% (w/v) agar, and the plates were placed at an angle of 70° in plant-growth chambers under light cycle (16-h light/8-h dark; 100 µmol/m^2^/s) conditions at 23 ± 1 °C. The length of the roots and shoots, number of lateral roots, and fresh weight were measured after 10 days of incubation. The most potential bacterial strains JBCE485 and JBCE486 were selected and further analyzed to determine the optimum concentrations to improve the growth of Arabidopsis. For co-inoculation, culture suspensions of each strain were mixed (1:1 v:v) and assessed for the growth promotion of Arabidopsis.

For analysis of salt stress alleviation activity, Arabidopsis seeds were treated with the optimum concentration of bacterial cells (1 × 10^7^ CFU/mL) of individual strains and combinations, and placed on ½ MS media supplemented with 100 mM NaCl. The plates were incubated under the same conditions as described above for 21 days, and the growth parameters were measured. The experiment consisted of three replicates with five seeds each, and the entire experiment was repeated twice.

### Growth promotion and salt stress alleviation assay of chive plant

Common chive (*Allium schoenoprasum* cv. Speed green), also known as onion chive, belonging to *Allium* sp. were used to asses growth promotion and stress resistance since U-SMN plants are generally propagated asexually and are difficult to grow under laboratory conditions. Seeds of chive were immersed in 70% ethanol for 90 s in Petri dishes, followed by 2% sodium hypochlorite (bleach) for 8 min, and thoroughly rinsed with sterile DW five times. The seeds were soaked in sterile DW for 24 h, then the seeds were treated with a cell suspension (1 × 10^7^ CFU/mL) of JBCE485, JBCE486, or a combination of JBCE485 + JBCE486 for 1 h. The treated seeds were sown in pots (5 seeds/pot) containing horticultural nursery soils that were soaked with 175 mM salt (soil/water, 2:1 v/v), and then incubated in a growth chamber at 20 ± 1 °C, 16-h light/8-h dark cycle for 4 weeks. The treated seeds were also sown in soil without salt treatment to assess the plant growth-promoting effect of the individual (JBCE485, JBCE486) and combination treatments. Shoot length, root length, and fresh weight of plants were measured 4 and 6 weeks after treatment in normal and salt-amended soil, respectively. Three independent experiments were performed, with at least 30 plants per treatment.

### Compatibility assay between JBCE485 and JBCE486

Each bacterial strain was grown on LB agar medium at 30 °C for 48 h. One strain (JBCE485) was streaked on fresh LB agar medium, and another (JBCE486) strain was streaked at an angle of 90° from the edge of the first strain streaking. The plates were incubated at 30 °C for 3 days and observed for inhibition zone. In addition, antagonism between JBCE485 and JBCE486 was assayed using a dual inoculation technique. Test plates were prepared with LB agar medium mixed with JBCE486 (1 × 10^7^ CFU/mL), and 20 μL cells of JBCE485 that were grown for 24 h were spot inoculated (1 × 10^7^ CFU/mL) on paper disks (8 mm). The plates were observed 2 days after incubation at 30 °C for inhibition zone. The experiment was replicated twice, with three plates per replicate.

### Assays for the plant growth promoting traits of bacteria

Siderophore production was assayed using the modified chrome azurol S (CAS) agar method^59^. Briefly, overnight grown individual (30 µL) and combined (15 µL each) bacterial cultures (1 × 10^7^ CFU/mL) in LB broth were spot inoculated onto sterile paper discs laid on CAS plates, and the plates were incubated at 30 °C for 4 days. Isolates exhibiting an orange halo were considered positive for siderophore production. Phosphate solubilization ability was determined by inoculating each and combined bacterial strain on a paper disc placed on Pikovskaya’s agar medium^60^. After 3 days of incubation at 30 °C, strains that induced a clear zone around the colonies were considered positive. Extracellular protease activity was determined by spot inoculation of individual and combined strains on sterile filter paper discs placed on skim milk agar medium^61^. Casein degradation, as indicated by clear zones, was measured 3 days after cultivation at 30 °C.

### Phytohormone production assay of bacteria

IAA production was determined by culturing bacterial strains in LB for 24 h at 30 °C before inoculating each strain or in combination into a medium containing l-tryptophan (5 mM) with a cell concentration of 0.5 at OD_600_ nm^62^. The cell-free supernatant of each combined culture was obtained by centrifugation after 48 h of incubation in the dark, mixed with Salkowski solution (3:2), and incubated in the dark. The IAA concentration was measured spectrophotometrically at 530 nm and quantified using a standard curve. For cytokinin production, each strain or combination was grown in M9 medium supplemented with 0.2% casamino acids, 0.01% thiamine, and 2 pg/L of biotin^63^ and measured spectrophotometrically at 665 nm^64^. The experiment was conducted in triplicate.

### Arabidopsis growth assay using phytohormone inhibitors and hormonal synthesis mutant

Arabidopsis (Col-0) seeds were bacterized with individual cell suspensions (1 × 10^7^ CFU/mL) of individual (JBCE485 or JBCE486) and combined strains and sown in MS supplemented with hormone inhibitors: 30 µM amino oxy acetic acid (30 µM auxin-), cycloheximide (cytokinin-10 µM), daminozide (gibberellins-20 µM), silver nitrate (ethylene-20 µM), and brassinosteroid (propiconazole-1 nM)^24^. The plates were cultivated as described above, and plant growth was photographed 14 days after treatment. Arabidopsis *eir1* (auxin transport-deficient and ethylene-insensitive) mutants were treated with bacterial cells and recorded, as described above.

### Population dynamics of co-inoculated strains in Arabidopsis and Chive

Seeds of Arabidopsis Col-0 dipped in the cell suspension (1 × 10^7^ CFU/mL) of individual (JBCE485 or JBCE486) and combined strains were sown on ½ MS medium and incubated in plant-growth chambers under light cycle (16-h light/8-h dark; 100 µmol/m^2^/s) conditions at 23 ± 1 °C, as described above. At 7 and 14 days, roots collected from 25 plants and populations of JBCE485 and/or JBCE486 on the surface of the roots were estimated using the serial dilution plating method. Because JBCE485 and JBCE486 were intrinsically resistant to rifampicin and tetracycline, respectively, the diluted suspensions were plated on LB medium containing rifampicin (25 µg/mL) and tetracycline (25 µg/mL) to count the number of JBCE485 and JBCE486 cells. The plates were incubated at 30 °C for 48 h, and the colony-forming units were calculated.

Seeds of chive plants were treated with individual or combined strains and sown in horticultural nursery soils. All tissues under the soil surface were collected 7 days after growth, and only roots were sampled at 14, 21, and 28 days after cultivation. The collected samples were rinsed with water, vortexed in 0.05 M phosphate buffer for 2 min, and the population per gram of tissue or root was evaluated by serial dilution plating as described above. To analyze bacterial cells that reside inside the roots of chive, collected samples were surface sterilized using 70% ethanol for 90 s, followed by 1% NaOCl for 8 min and five washes with sterile DW. The roots were macerated with 0.05 M phosphate buffer and serially diluted samples were plated as described above. The experiment was replicated twice, with 15 seedlings per replicate.

### Statistical analysis

The procedures for statistical analysis of the bacterial community diversity assays are described in each section. Plant growth analysis experiments were done in completely randomized design and the data were subjected to one-way analysis of variance using the SAS JMP software (SAS Institute, Cary, NC, USA). The data was first tested for normality using Kolmogorov–Smirnov test and homogeneity of variance with the Brown–Forsythe test. Significant differences were then determined in the treatment means of each sample using the least significant difference (LSD) test at *P* = 0.05. Data from each experiment were analyzed separately. The population of bacterial cells colonizing the surface and inside the roots was analyzed using the Student’s *t*-test at P = 0.05.

## Supplementary Information

Supplementary Information.

## Data Availability

The datasets generated and analysed during the current study are available in the National Center for Biotechnology Information (NCBI) repository, and the web links are as follows: https://www.ncbi.nlm.nih.gov/sra/PRJNA831589, https://www.ncbi.nlm.nih.gov/sra/PRJNA831594, https://www.ncbi.nlm.nih.gov/sra/PRJNA831599, https://www.ncbi.nlm.nih.gov/sra/PRJNA831600, https://www.ncbi.nlm.nih.gov/sra/PRJNA831604, and https://www.ncbi.nlm.nih.gov/sra/PRJNA831611.
