# Supplementary material for: Characteristics of rhizosphere and endogenous bacterial community of Ulleung-sanmaneul, an endemic plant in Korea: application for alleviating salt stress
Source: Sci Rep. 2022 Dec 7;12:21124. doi: 10.1038/s41598-022-25731-z (PMC9729608; doi:10.1038/s41598-022-25731-z)
Supplement: Supplementary file 1 — Supplementary Information. [file 41598_2022_25731_MOESM1_ESM.pdf]

Characteristics of rhizosphere and endogenous bacterial community of Ulleung-sanmaneul, an endemic plant in Korea: Application for alleviating salt stress

Swarnalee Dutta[1], Yeong-Su Kim[2], Yong Hoon Lee[1,3,*]

[1]*Division of Biotechnology, Jeonbuk National University, 79 Gobong-ro, Iksan-si, Jeollabuk-do 54596 Republic of Korea*

[2]*Wild Plants and Seeds Conservation Department, Baekdudaegan National Arboretum, Bonghwa-gun, Gyeongsangbuk-do, 36209, Republic of Korea*

[3]*Advanced Institute of Environment and Bioscience, Plant Medical Research Center, and Institute of Bio-Industry, Jeonbuk National University, Jeonju-si, Republic of Korea*

*Author for Correspondence:
Tel: +82-63-850-0841; Fax: +82638500834; E-mail: yonghoonlee@jbnu.ac.kr

Supplementary data

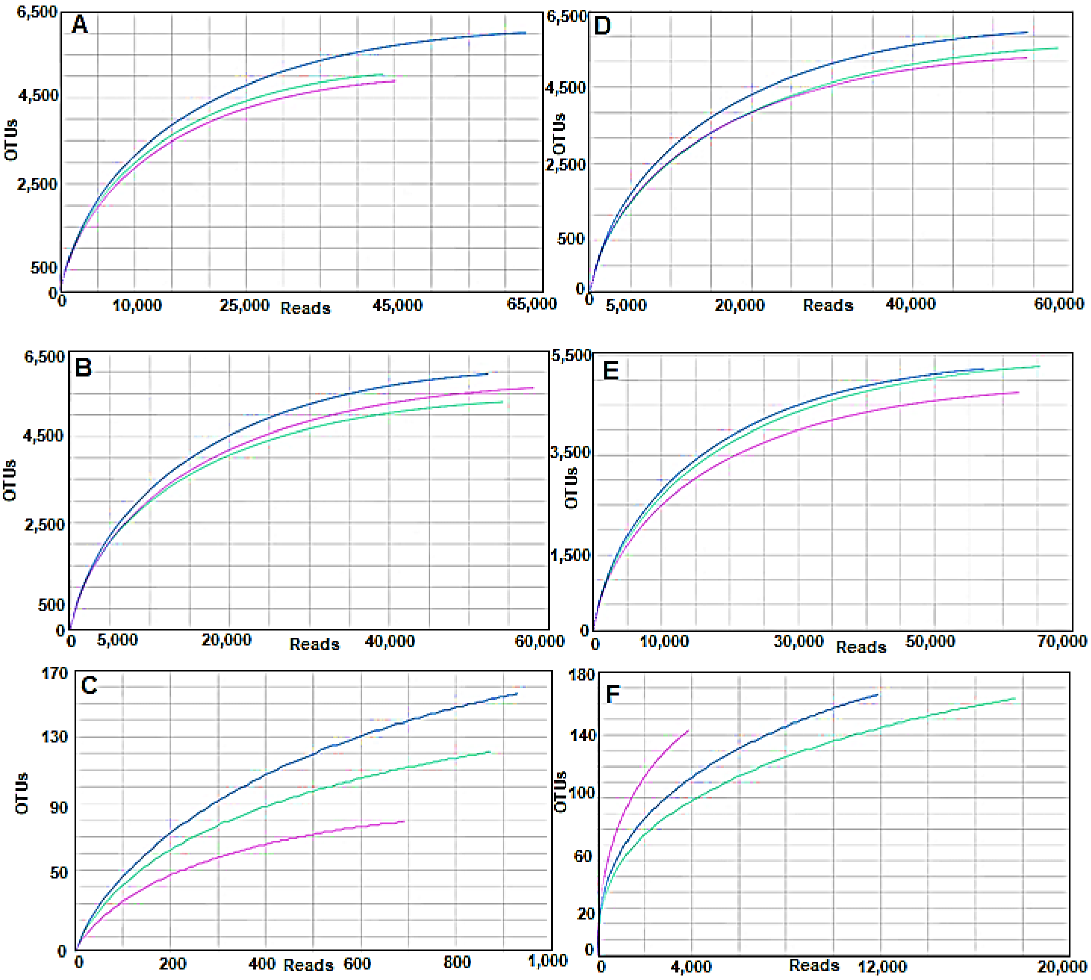

Supplementary Figure S1. Rarefaction curves for bacterial operational taxonomic units (OTUs) of Ulleung-sanmaneul plant. Rarefaction curves of bulk soil (A, D), rhizosphere (B, E), and endophyte (C, F) in wild and cultivated area, respectively with cut-off value at 97% similarity. The vertical axis indicates the number of OTUs expected after sampling the number of sequences denoted in the horizontal axis.

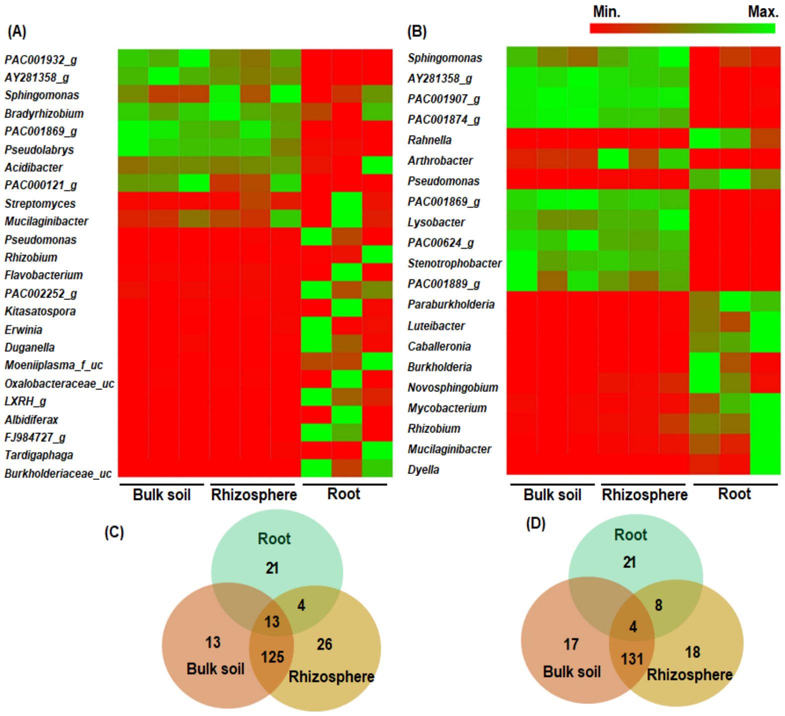

Supplementary Figure S2. Heat map analysis to compare the overall bacterial profile in different rhizocompartments of Ulleung-sanmaneul plants. The bacterial genera with more than 1% relative abundance in each compartment of wild (A) and cultivated (B) habitats were selected for comparison. The Venn diagrams show unique or shared genera of bacterial microbiota with relative abundance above 0.1% in each compartment of wild (C) and cultivated (D) areas.

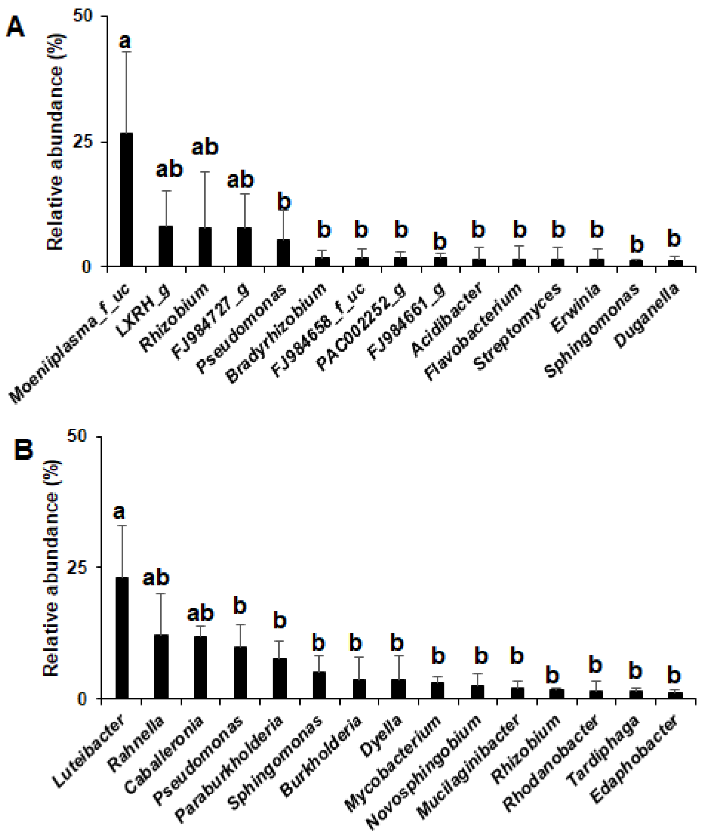

Supplementary Figure S3. Relative abundance of bacterial genera in roots of Ulleung-sanmaneul plants. Top 15 bacterial genera with relative abundance >1% in roots of wild (A) and cultivated (B) plants were compared. The data are represented as mean ± standard deviation and bars with the same letter do not differ significantly at *P*=0.05.

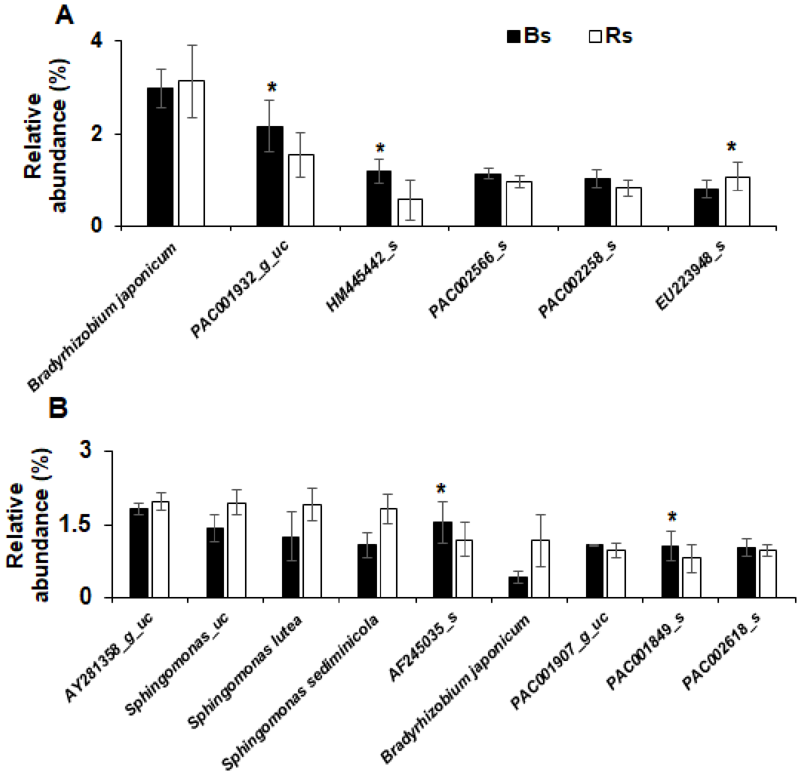

Supplementary Figure S4. Relative abundance of bacterial species in bulk soil and rhizosphere of Ulleung-sanmaneul plants. The bacterial genera with relative abundance >1% in at least one compartment of bulk soil and rhizosphere in wild (A) and cultivated (B) habitats were compared. Data represent mean ± standard deviation and values marked by an asterisk (*) are significantly different between compartments in respective habitats.

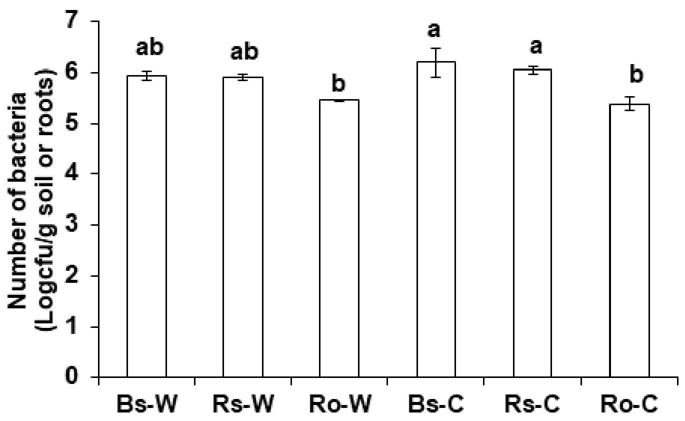

Supplementary Figure S5. Total number of culturable bacteria in bulk soil, rhizosphere, and root of Ulleung-sanmaneul plant. Bacterial colonies were assessed from bulk soil (Bs), rhizosphere soil (Rs), and root (Ro) of plants collected from wild (-W) and cultivated (-C) area. Data presented as mean±SD of log cfu per g soil or roots and bars with same letter do not differ significantly at P=0.05.

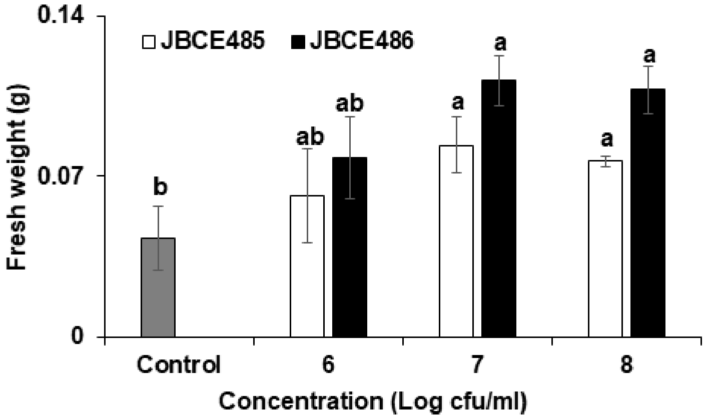

Supplementary Figure S6. Effect of different concentrations of *Pseudoxanthomonas* sp. JBCE485 and *Variovorax paradoxus* JBCE486 on the growth of Arabidopsis. Seeds of *Arabidopsis thaliana* Col-0 were treated with 1×10^6, 10^7, and 10^8 cfu/ml bacterial suspension of each bacterium and cultivated on half-strength MS medium. The growth performance was analyzed by measuring fresh weight of five plants. Data represent mean±SD and bars with same letter do not differ significantly at P=0.05 for individual bacteria.

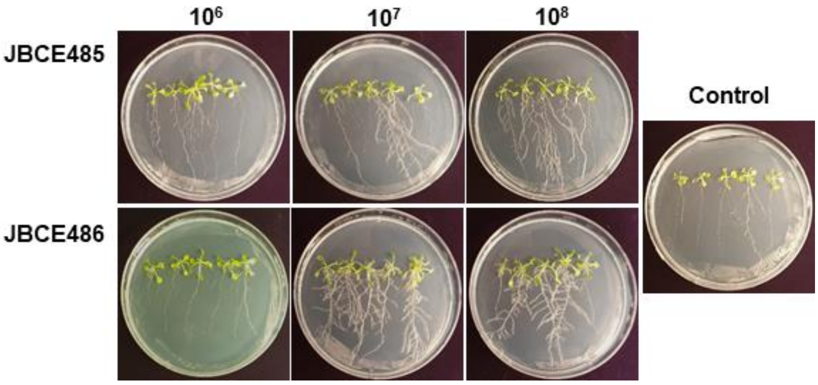

Supplementary Figure S7. Growth promotion of Arabidopsis by treatment of various cell concentrations of *Pseudoxanthomonas* sp. JBCE485 and *Variovorax paradoxus* JBCE486. Seeds of *Arabidopsis thaliana* Col-0 were treated with 1×10^6, 10^7, and 10^8 cfu/ml bacterial suspension of each bacterium and cultivated on half-strength MS medium. The photos were taken 14 days after treatment.

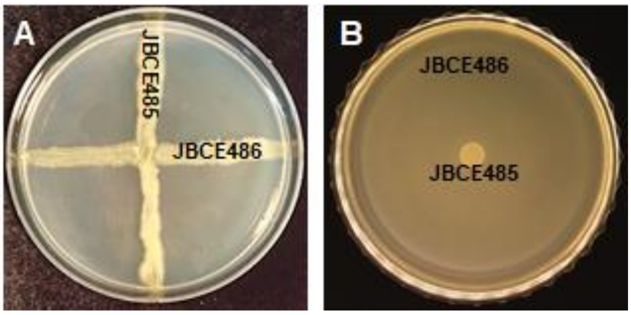

Supplementary Figure S8. Compatibility between *Pseudoxanthomonas* sp. JBCE485 and *Variovorax paradoxus* JBCE486. (A) Both strains were streaked perpendicularly as indicated in the photo. (B) One strain (JBCE486) was mixed with LB media and another strain (JBCE485) was inoculated on the paper disk. Growth inhibition or inhibition zone was photographed 3 days after incubation.

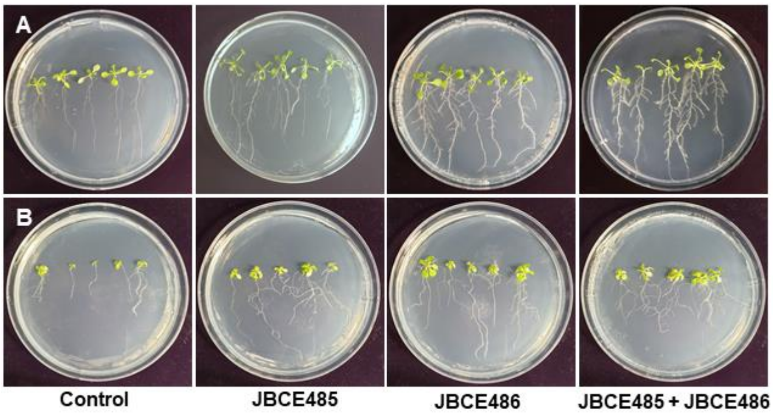

Supplementary Figure S9. Growth promotion and salt stress alleviation of Arabidopsis by treatment with *Pseudoxanthomonas* sp. JBCE485 and *Variovorax paradoxus* JBCE486. Seeds of *Arabidopsis thaliana* Col-0 were treated with 1×10^7 CFU/mL cells of individual and combined strains of JBCE485 and JBCE486. Seeds were grown on ½ MS medium (A) without and (B) with 100 mM NaCl.

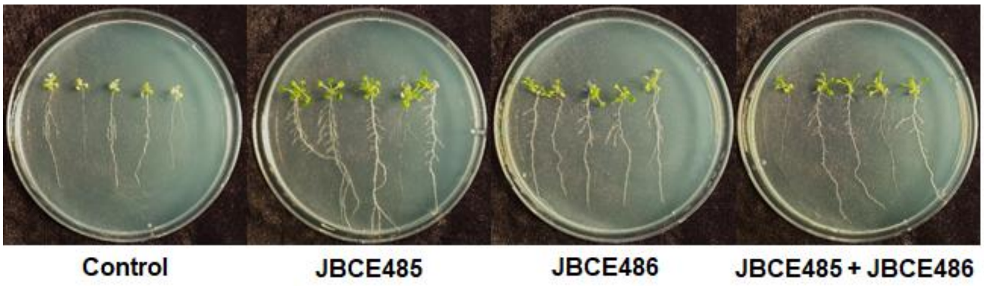

Supplementary Figure S10. Effect of phytohormone inhibitors on growth of Arabidopsis by treatment of *Pseudoxanthomonas* sp. JBCE485 and *Variovorax paradoxus* JBCE486. Seeds of *Arabidopsis thaliana* Col-0 were treated with 1×10^{7} cfu/ml cells of individual and combined strains and placed on half-strength MS medium amended with hormone inhibitor aminooxyacetic acid. Arabidopsis growths were recorded two weeks after incubation.

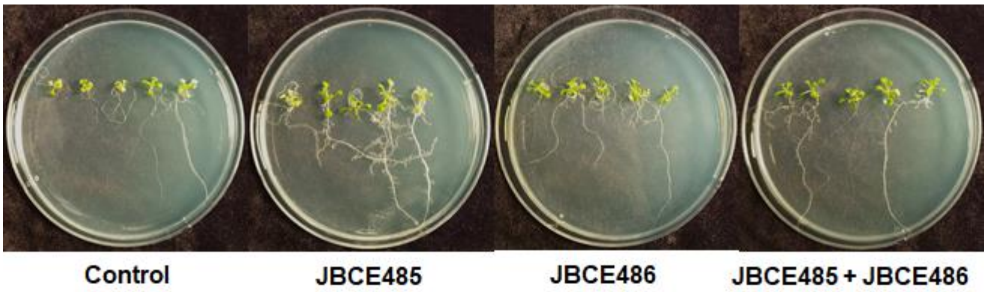

Supplementary Figure S11. Effect on growth of Arabidopsis mutant by treatment of *Pseudoxanthomonas* sp. JBCE485 and *Variovorax paradoxus* JBCE486. Seeds of *Arabidopsis thaliana* Eir-1 were treated with 1×10^{7} cfu/ml cells of individual and combined cells of JBCE485 and JBCE486 and placed on half-strength MS medium for two weeks.

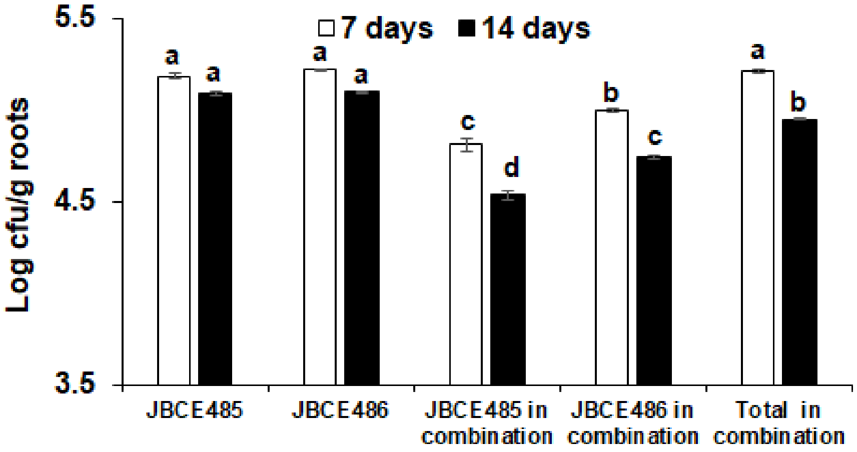

Supplementary Figure S12. Population dynamics of *Pseudoxanthomonas* sp. JBCE485 and *Variovorax paradoxus* JBCE486 cells on Arabidopsis roots. Seeds of *Arabidopsis thaliana* Col-0 were treated with individual and combined cell suspension (1×10^7 cfu/ml) of JBCE485 and JBCE486 and sown on half-strength MS medium. Root samples taken at 7 and 14 days after cultivation were vortexed for 1 min in 0.05 M phosphate buffer and plated on LB medium with rifampicin (25 µg/ml) and tetracycline (25 µg/ml) for JBCE485 and JBCE486, respectively. After incubation at 30°C for 48 h, the colonies were counted and their number per g roots were calculated. The experiment was replicated three times with 25 seedlings per replicate. The data are represented as standard deviation of 5 independent biological samples. Bars with same letter(s) do not differ significantly at P=0.05 at each timepoint

Supplementary Table S1. Physiochemical properties of soils around Ulleung-sanmaneul plants

Area (Replication)	Properties					
	pH	Electrical conductivity (dS/m)	Ca^{2+} (cmol/Kg)	K^{+} (cmol/Kg)	Mg^{2+} (cmol/Kg)	Na^{+} (cmol/Kg)
Wild area						
1	5.93±0.02	0.54±0.03	4.98±0.12	0.49±0.02	2.69±0.06	1.29±0.08
2	5.92±0.01	0.38±0.01	3.23±0.05	0.47±0.03	1.95±0.00	1.40±0.02
3	6.01±0.02	0.43±0.02	3.78±0.05	0.64±0.00	3.15±0.05	0.78±0.10
Cultivated area						
1	6.83±0.02	0.18±0.01	8.17±0.10	0.81±0.01	2.46±0.03	3.96±0.00
2	6.87±0.03	0.18±0.04	8.46±0.00	0.79±0.01	2.31±0.04	3.79±0.06
3	6.92±0.02	0.18±0.01	8.26±0.08	0.94±0.01	2.62±0.03	3.60±0.04

Supplementary Table S2. Estimated OTU richness and diversity indices in the bulk soil, rhizosphere soil, and roots from plants in wild area

Sample		Valid reads	OTUs	Ace	Chao1	JackKnife	Shannon	Simpson	Goods Library Coverage (%)
Bulk soil	1	43,527	5,048	5,362.33	5,173.84	5,667	7.4531	0.00185	98.5779
	2	62,722	6,022	6,254.55	6,110.62	6,550	7.5922	0.00155	99.1582
	3	45,207	4,893	5,182.17	5,012.69	5,472	7.3317	0.00229	98.7192
Rhizosphere soil	1	54,275	5,304	5,560.93	5,415.16	5,857	7.4651	0.00207	98.9811
	2	52,397	5,955	6,282.86	6,081.27	6,622	7.6074	0.00209	98.7270
	3	58,057	5,635	5,918.49	5,757.66	6,235	7.5050	0.00178	98.9665
Root	1	872	121	165.29	156.88	165	3.6031	0.06633	95.1835
	2	929	156	212.83	198.78	213	3.9500	0.04316	93.9720
	3	690	79	93.38	86.92	99	2.8799	0.14478	97.1015

Supplementary Table S3. Estimated OTU richness and diversity indices in the bulk soil, rhizosphere soil, and roots from plants in cultivated area

Sample		Valid reads	OTUs	Ace	Chao1	JackKnife	Shannon	Simpson	Goods Library Coverage (%)
Bulk soil	1	58,026	4,768	5,038.71	4,887.63	5318	7.0542	0.00329	99.0522
	2	54,260	5,076	5,329.32	5,175.66	5604	7.3041	0.00220	99.0269
	3	54,161	4,573	4,804.39	4,667.93	5052	7.1465	0.00247	99.1156
Rhizosphere soil	1	65,453	5,275	5,554.49	5,400.55	5856	7.0559	0.00525	99.1123
	2	57,210	5,225	5,511.11	5,356.35	5823	7.2243	0.00320	98.9547
	3	62,333	4,758	5,055.03	4,907.33	5342	6.912	0.00547	99.0631
Root	1	177,12	163	214.30	193.94	208	2.6491	0.13726	99.7459
	2	11,899	166	227.25	213.36	221	2.8352	0.11255	99.5630
	3	3,874	143	192.56	197.05	223	3.0474	0.09997	98.7868

Supplementary Table S4. Comparison of top 25 endophytic bacterial species in roots of wild and cultivated plants

Wild plant	Relative abundance (%)±SD	Cultivated plant	Relative abundance (%)±SD
FJ984727_s	6.6±5.8	*Luteibacter rhizovicinus*	23.0±9.6
Rhizobium cellulosilyticum	5.6±9.5	*Rahnella aquatilis*	12.2±7.8
LXRH_g_uc	5.0±3.3	*CP014505_s*	11.8±1.8
Pseudomonas_uc	3.3±3.9	*Pseudomonas fulva*	6.4±3.6
JN038929_s	2.3±2.6	*Dyella koreensis*	3.4±4.6
Bradyrhizobium japonicum	2.0±1.4	*Paraburkholderia caledonica*	2.7±3.5
FJ984661_s	1.6±0.7	*Novosphingobium rosa*	2.6±2.3
Streptomyces scabiei	1.5±2.3	*Sphingomonas pruni*	2.4±0.2
Erwinia billingiae	1.4±2.2	*Burkholderia pyrrocinia*	2.4±4.0
Flavobacterium saccharophilum	1.4±2.5	*Paraburkholderia dipogonis*	2.1±1.8
PAC001319_s	1.2±1.7	*Mycobacterium hodleri*	1.9±0.6
FNJA_s	1.1±2.0	*Rhizobium leguminosarum*	1.6±0.2
Pseudomonas fulva	1.0±1.4	*Pseudomonas viridiflava*	1.4±1.0
EU335421_s	1.0±0.4	*Rhodanobacter denitrificans*	1.4±1.8
FJ984727_g_uc	1.0±1.1	*Tardiphaga robiniae*	1.4±0.8
Streptomyces avellaneus	0.9±1.4	*Burkholderia cepacia*	1.3±1.3
Rhizobium mongolense	0.9±1.6	*BACX_s*	1.2±1.8
Mycobacterium abscessus	0.9±0.6	*Paraburkholderia sediminicola*	1.2±0.9
PAC002252_g_uc	0.8±1.0	*Pseudomonas marginalis*	1.1±1.1
Rhizobium tibeticum	0.8±0.9	*Mycobacterium llatzerense*	0.9±0.3
EF516303_s	0.8±0.7	*Edaphobacter modestus*	0.7±0.5
FJ984676_s	0.7±1.3	*Bradyrhizobium japonicum*	0.7±0.4
FJ984716_g_uc	0.7±1.3	*FNJV_s*	0.7±1.0
FR687494_s	0.7±1.2	*Paraburkholderia graminis*	0.7±0.2
Tardiphaga robiniae	0.7±1.2	*Sphingomonas polyaromaticivorans*	0.7±0.9

Supplementary Table S5. Characteristics related with plant growth promoting activities of *Pseudoxanthomonas* sp. JBCE485 and *Variovorax paradoxus* JBCE486

Strains	Production or activity				
	Siderophore production	Phosphate Solubilization	Protease activity	IAA production (μg/ml)	Cytokinin production
Pseudoxanthomonas sp. JBCE485	+[a]	-	+	++	-
Variovorax paradoxus JBCE 486	+	-	-	+	-
JBCE485 + JBCE486	+	-	+	+++	-

[a]+++; very high, ++; high, +; low, -; no production or activity
